# Periodic Therapeutic Phlebotomy Mitigates Systemic Aging Phenotypes by Promoting Bone Marrow Function

**DOI:** 10.1111/acel.70400

**Published:** 2026-02-02

**Authors:** Ji‐Ru Cai, Jian Zhang, Yue‐Xin Ning, Jing Zhang, Tian‐Ce Xu, Mei‐Chen Liu, Ke‐Xin Wang, Hui‐Sheng Chen

**Affiliations:** ^1^ Department of Neurology General Hospital of Northern Theater Command Shenyang China

## Abstract

Aging is the primary risk factor for numerous chronic diseases, making the identification of safe and effective anti‐aging strategies a critical focus in biomedical research. Heterochronic parabiosis by blood exchange shows that the exchange interaction between young and old plasma can exert anti‐aging effects through exchange of bloodborne factors. However, the limited plasma source greatly affects clinical translation. Here, we demonstrate that periodic therapeutic phlebotomy in D‐galactose‐induced aging models exerts significant and comprehensive anti‐aging effects, which is reflected by a notable improvement in aging‐associated behavioral deficits and neurogenesis, a significant decrease in the level of circulating senescence‐associated secretory phenotypes, and an obvious mitigation of aging‐associated structural degradation and molecular alterations within the muscle, bone, liver, kidney, and nervous systems. Mechanistically, periodic therapeutic phlebotomy induces bone marrow microenvironment restoration through functional rescue of mesenchymal stem cells and endothelial cells, thereby reestablishing balanced hematopoietic homeostasis. This hematopoietic revitalization subsequently drives systemic improvements in peripheral blood composition and function. In conclusion, our work provides preliminary evidence suggesting that periodic therapeutic phlebotomy exerts anti‐aging effects by restoring bone marrow function and mitigating aging phenotypes, subsequently driving peripheral blood functional restoration. Given its technical simplicity and safety profile, this periodic therapeutic phlebotomy strategy will hold potential to pave the way for clinical translation.

## Graphical abstract

In D‐galactose‐induced aging model, periodic therapeutic phlebotomy exerts significant and comprehensive anti‐aging effects by inducing bone marrow microenvironment restoration, which rebalances hematopoiesis to restore peripheral blood function and ameliorate systemic aging phenotypes. This finding provides preliminary evidence suggesting anti‐aging effects of periodic therapeutic phlebotomy.

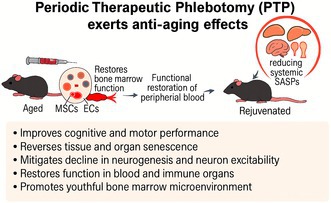

## 1 Introduction

As a multifactorial and complex biological process, aging affects the systematic degeneration of various tissues and organs throughout the body, resulting in a progressive decline in the body's regenerative capacity and overall functional abilities. Aging is characterized by chronic inflammation throughout the body, accompanied by cellular senescence, immune senescence, organ dysfunction and aging‐related diseases (Arai et al. 2015; López‐Otín et al. 2023; Sayed et al. 2021; Yousefzadeh et al. 2021). Interventions based on young factors have demonstrated considerable potential in counteracting aging phenotypes. For example, infusion of young cerebrospinal fluid into aged mice could improve memory function (Iram et al. 2022), while multiple elegant studies about heterochronic parabiosis and young plasma transfer can rejuvenate old tissues and aging‐related molecular phenotype and behavior (Carlson et al. 2009; Conboy et al. 2005; Loffredo et al. 2013; Sinha et al. 2014; Villeda et al. 2014; Jeon et al. 2022). It has been reported that young blood has the potential to reverse senescence‐associated structural deterioration and molecular alterations in the muscular, skeletal, hepatic, and nervous systems (Lagunas‐Rangel 2024; Liu et al. 2024; Zhang et al. 2023).

During aging, there are significant alterations in the bone marrow niche, which subsequently impact both the quality and quantity of hematopoietic stem cells (HSCs) (Kuribayashi et al. 2021). The bone marrow endosteal niche undergoes degeneration and remodeling, which comes with a substantial loss of bone density due to reduced numbers of osteoblasts and a loss of mesenchymal stromal cell (MSC) differentiation toward the osteoblastic lineage. Decreased angiogenesis increases vascular leakiness, and attrition of arterioles further exacerbates the functional decline of the endosteal niche (Poulos et al. 2017; Stucker et al. 2020). These modifications culminate in impaired HSC functionality, a predisposition toward myeloid lineage differentiation, and a decline in regenerative capacity. Additionally, the aged niche is characterized by a pro‐inflammatory milieu with elevated levels of cytokines, which can directly compromise HSC expansion and self‐renewal capabilities (Cain et al. 2025; Frisch et al. 2019; Pietras 2017). As the fundamental basis of the hematopoietic system in living organisms, HSCs are crucial not only for the generation of erythrocytes, leukocytes, and thrombocytes but also as a significant source of peripheral immune cells (Colom Díaz et al. 2023; Nakamura‐Ishizu et al. 2020). According to the U.S. Clinical Trials Database, results are now available showing that stem cell transplants have demonstrated safe and effective properties in slowing down the aging process (Garay 2023). A recent study demonstrates that peripheral immune cells can be rejuvenated through transplanting young bone marrow cells in aged APP/PS1 mice thereby improving Alzheimer's disease‐like pathologies and behavioral deficits (Sun et al. 2024). Moreover, antibody‐mediated depletion of HSCs with myeloid‐biased in aged mice restores characteristic features of a more youthful immune system (Ross et al. 2024). Collectively, these studies suggest that restoring the functional capacity of hematopoietic stem cells in the bone marrow niche and reducing senescence markers are key pathways for mitigating systemic aging phenotypes.

Ischemic or hypoxic pre‐conditioning was found to enhance the mobilization and recruitment of bone marrow stem cells (Kamota et al. 2009; Yu et al. 2016). Similar to ischemic or hypoxic pre‐conditioning, acute blood loss also promoted proliferation of bone marrow (Lippiello et al. 1992; Sulc et al. 1976). Confronting the stress of acute blood loss, the bone marrow activates an emergency differentiation program, resulting in significant increases or decreases in the production of specific blood cell lineages (Wu et al. 2024). The process is followed by a return to homeostasis once the stress is removed (Swann et al. 2024; Mitroulis et al. 2018). In a clinical context, donors with regular blood donation were found to exhibit some benefits including reduced blood pressure, improved lipid profiles, reduced risk of certain types of cancers, and decreased risk of cardiovascular diseases (Gasparovic Babic et al. 2024). In this context, we hypothesized that a periodic and non‐harmful blood loss stimulation, namely periodic therapeutic phlebotomy (PTP), may stimulate the mobilization and recruitment of bone marrow stem cells to induce the youthfulness of the bone marrow hematopoietic system. This process is expected to maintain the youthfulness of the peripheral blood system including reducing senescence‐associated secretory phenotypes (SASPs), ultimately ameliorating age‐related decline in various tissues and organs.

The D‐galactose (D‐gal)–induced aging model is widely used for aging research in animals due to its ability to mimic several key aspects of natural aging (Shwe et al. 2018; Ho et al. 2003). Chronic administration of D‐gal leads to aging‐like changes, including increased oxidative stress, neuronal degeneration, inflammation, and apoptosis, which are similar to those observed in natural aging processes in both animals and humans (Ma et al. 2024; Rajendran et al. 2024; El‐Far et al. 2024). In this study, we tested this hypothesis in D‐galactose‐induced aging rats and mice.

## 2 Results

### 2.1 PTP Improves Cognitive and Motor Performance Associated With Aging

**FIGURE 1:**
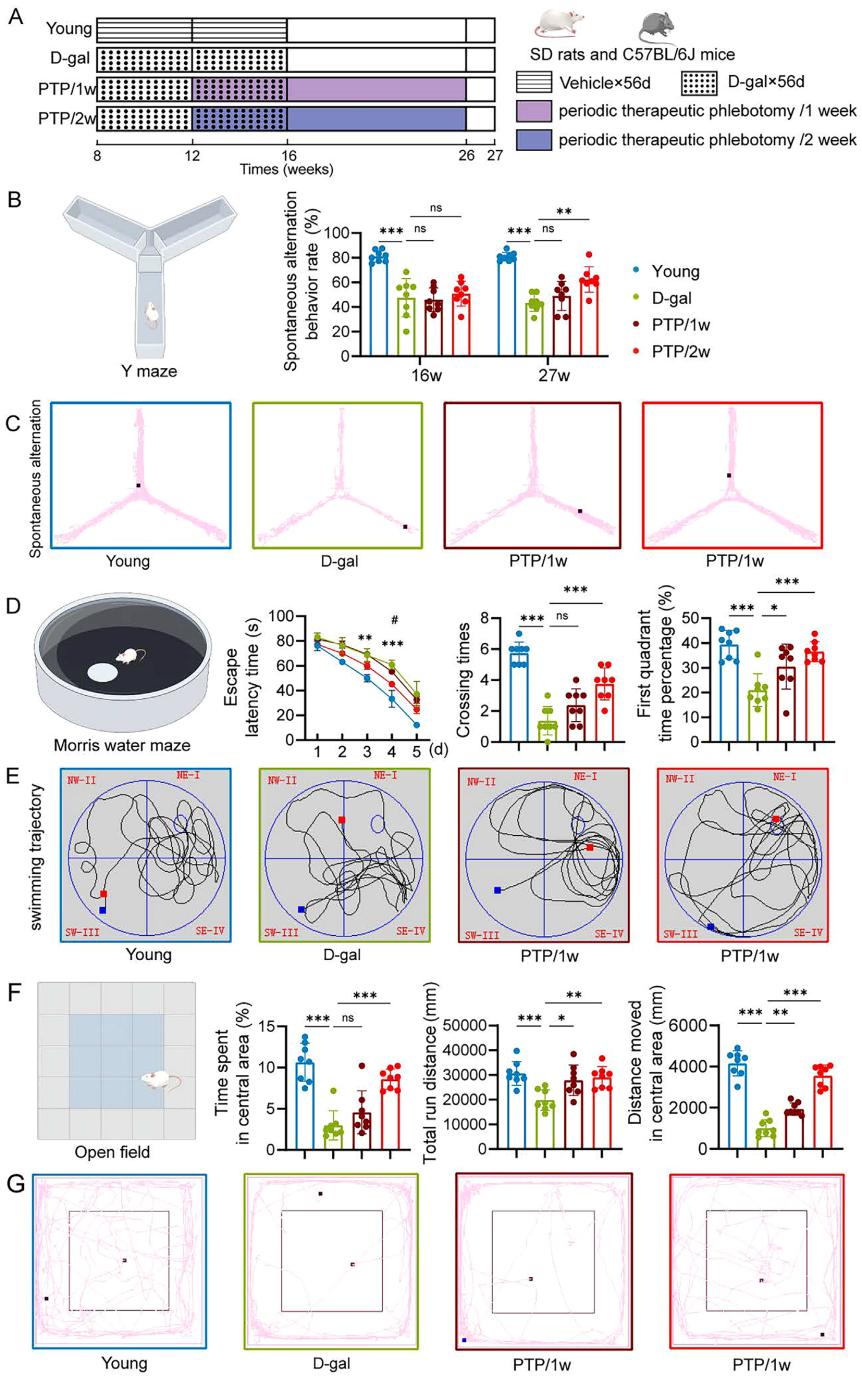
PTP improves cognitive and motor performance. (A) The intervention flowchart of SD rats. (B) Diagram of Y maze, and spontaneous alternation behavior rate of rats in Y maze. (C) Representative moving trajectory of Y maze of rats on the 26th week. (D) Diagram of Morris water maze, and escape latency time, crossing times, first quadrant time percentage in Morris water maze. (E) Representative moving trajectory of Morris water maze of rats. (F) Diagram of open field, and time spent in central area, total run distance, and distance moved of rats in open field. (G) Representative moving trajectory in open field of rats. *n* = 8/group. Statistical analysis: Two‐way ANOVA and Tukey post‐test for (D) escape latency time and one‐way ANOVA and Tukey post‐test for others. Data are mean ± SD; error bars denote 95% CI; *p < 0.05, **p < 0.01, ***p < 0.001.

To determine the anti‐aging effects of PTP, we utilized the D‐galactose‐induced aging model in both SD rats and C57BL/6J mice. The PTP intervention was performed throughout the entire aging period (Figure 1A).

Cognitive alterations naturally arise throughout the aging process in mammals evidenced by a deterioration in learning and memory functions. We employed the Y‐maze and Morris water maze to assess cognitive function, the open field test to evaluate general locomotor activity and anxiety‐like behavior, the treadmill test to measure motor endurance and exercise capacity, and the tail suspension test (TST) and forced swim test (FST) to assess depression‐like behaviors in rats and mice across different groups.

To assess the potential of PTP in enhancing cognitive and motor performance in D‐galactose‐induced aged rats, the working memory capacity of the rats was assessed by two Y‐maze spontaneous alternation tests administered on 16th and 26th week. The experimental results indicated a significant reduction in spontaneous alternating behaviors in the D‐gal group compared to the Young group, which was significantly improved in the PTP/2w group, but not in the PTP/1w group (Figure 1B,C). The same results were also observed in the experiments with mice (Figure S1A,B). We employed the Morris water maze test to evaluate spatial learning and memory (Figure 1D). A parallel experiment was conducted on mice to corroborate these findings (Figure S1C). The escape latency time in the D‐gal group was significantly higher compared to the Young group, which was significantly mitigated by PTP with a more pronounced effect in the PTP/2w group vs. the PTP/1w group (Figure 1D). The same results were also observed in the experiments with mice (Figure S1D). The number of crossing times was significantly lower in the D‐gal group compared to the Young group, which was significantly reversed in the PTP/2w group, but not in the PTP/1w group (Figure 1D). The same results were also observed in the mice (Figure S1D). The percentage of time spent in quadrant I was significantly lower in the D‐gal group vs. the Young group. The PTP effectively reversed this trend, with the PTP/2w group showing greater improvement compared to the PTP/1w group (Figure 1D). The same results were also achieved in mice (Figure S1D). We utilized the open field test to assess the autonomous locomotor abilities and anxiety levels in rats. Compared with the Young group, the total running distance was significantly less in the D‐gal group, which was significantly reversed in PTP groups, with the PTP/2w group outperforming the PTP/1w group (Figure 1F). Similar findings were found regarding the distance moved and the time spent in the central area (Figure 1F). The same results were also observed in the mice (Figure S1E).

We utilized the tail suspension test to assess depression‐like behaviors. The significantly lower performance was found in the D‐gal group compared to the Young group in both hanging time and hanging endurance metrics, which was significantly improved in the PTP/2w group (Figure S1I,J). Similar trends were observed in the exhaustion time during the treadmill experiment (Figure S1G,H) and the forced swim test (Figure S1K,L).

Collectively, these findings suggest that the PTP significantly improves cognitive and motor functions in D‐galactose‐induced aging models of both rats and mice, with a greater effect in the PTP/2w than the PTP/1w group.

### 2.2 PTP Reverses the Senescence of Tissues and Organs Associated With Aging

In this experiment, we investigate the effect of PTP on the senescence of tissues and organs.

First, an analysis of multiple tissues using RT‐qPCR demonstrated that in the hippocampus, cortex, heart, liver, kidneys, and skeletal muscles (gastrocnemius and tibialis anterior) of D‐galactose‐induced aged rats, there was a significant elevation in the mRNA expression levels of CDKN2A and CDKN1A, as well as several senescence‐associated secretory phenotype (SASP) components, including IL‐6, P21, IL‐1β, and TNF‐α, compared to the Young group (Figure S2aA). The increases were significantly reduced by PTP, with a greater effect in the PTP/2w group than the PTP/1w group. However, no significant changes were observed in the lung (Figure S2aA). The findings suggest that PTP has the potential to reduce SASP levels in multiple tissues, thereby mitigating the accumulation of systemic chronic inflammation. Furthermore, we observed the protein changes of SASP factors in the skeletal muscle and hippocampus given their key role in aging. Numerous SASP‐related factors, such as IL‐1α, IL‐6, CCL5, and TNF‐α, exhibited significantly higher concentrations in the hippocampus (Figure S2aB) and tibialis anterior (Figure S2aC) of D‐galactose‐induced aged rats compared to young rats. These changes were significantly reversed by PTP with a greater effect in the PTP/2w group than the PTP/1w group.

**FIGURE 2:**
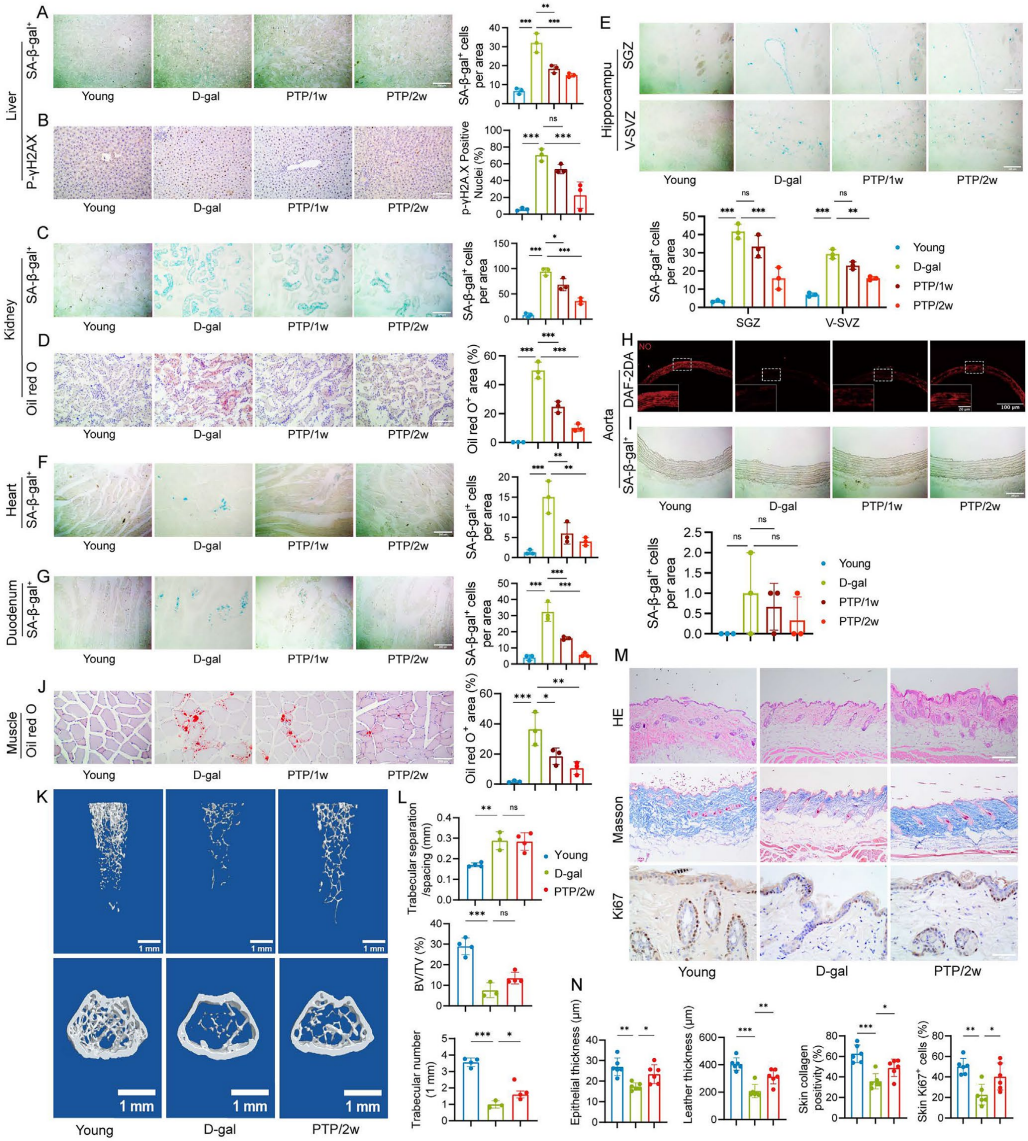
PTP mitigates the senescence of tissues and organs. (A) Representative SA‐β‐gal staining of rat liver (scale bars, 200 μm), and quantification of SA‐β‐gal^+^ cells per area, *n* = 3/group. (B) Representative p‐γH2A.X staining of rat liver (scale bars, 200 μm), and quantification of p‐γH2A.X positive nuclei percentage, *n* = 3/group. (C) Representative SA‐β‐gal staining of rat kidney (scale bars, 200 μm), and quantification of SA‐β‐gal^+^ cells per area, *n* = 3/group. (D) Representative Oil red O staining of rat kidney (scale bars, 200 μm), and quantification of Oil red O^+^ area, *n* = 3/group. (E–G) Representative SA‐β‐gal staining in hippocampus (SGZ and V‐SVZ), heart, and duodenum of rat (scale bars, 200 μm), and percentage of SA‐β‐gal^+^ cells per area, *n* = 3/group. (H) Representative DAF2DA fluorescence staining for NO levels in aortas (scale bars, 100 μm). (I) Representative SA‐β‐gal staining of rat aorta (scale bars, 200 μm), and quantification of SA‐β‐gal^+^ cells per area, *n* = 3/group. (J) Representative Oil red O staining of rat skeletal muscle (scale bars, 200 μm), and quantification of Oil red O^+^ area, *n* = 3/group. (K) Representative micro‐CT in murine femur (scale bars, 1 mm). (L) Bone tissue volume/total tissue volume (BV/TV), trabecular number (Tb·N), and trabecular separation/spacing (Tb. Sp) of bone tissue in each group were assessed by micro‐CT quantitative analysis, *n* = 3–4/group. (M) Representative of HE, Masson's staining and Ki67 immunohistochemistry staining of murine skin (scale bars, 400 μm). (N) Quantification of epithelial thickness, leather thickness, collagen positivity percentage and Ki67^+^ cells of skin, *n* = 6/group. Statistical analysis: one way ANOVA and Bonferroni post‐test for (L) and Tukey post‐test for the others. Data are mean ± SD; error bars denote 95% CI; *p < 0.05, **p < 0.01, ***p < 0.001.

Second, we explored the pathological alterations in the liver of rats. The results showed that β‐galactosidase (SA‐β‐gal) positive cells were significantly increased in the D‐gal group compared to the Young group (Figure 2A). Then, we used immunohistochemistry of p‐γH2A.X to evaluate liver DNA damage and found that DNA damage was more serious in the D‐gal group compared to the Young group (Figure 2B). To assess the hepatic lipidosis and fibrosis, we used Oil red O staining (Figure S2bA), Sirius red staining (Figure S2bB), and Desmin immunohistochemistry (Figure S2bC) and measured the expression of T‐BIL (Figure S2bD) and ALT (Figure S2bE) in rats. The results showed that the hepatic lipidosis and fibrosis were more serious in the D‐gal group compared to the Young group, which was mitigated by PTP, with greater effect in the PTP/2w group than the PTP/1w group (Figure 2B; Figure S2bA–E).

Third, we explored the pathological alterations in the kidney of rats. The results showed that SA‐β‐gal^+^ cells were significantly increased in the D‐gal group, compared to the Young group (Figure 2C). We used Oil red O staining to evaluate renal lipidosis and found that its amount was greater in the D‐gal group compared to the Young group (Figure 2D). Given the key role of HMGB1, BUN, and creatinine in renal damage, we also performed HMGB1 immunohistochemistry (Figure S2bF) and measured the expression of BUN (Figure S2bG) and creatinine (Figure S2bH) in rats. The results indicated that these biomarkers significantly increased in the D‐gal group compared to the Young group. These alterations above associated with kidney aging were significantly mitigated by PTP, with a greater effect in the PTP/2w group than the PTP/1w group (Figure 2C,D, Figure S2bF–H).

Fourth, we observed SA‐β‐gal^+^ cells in the hippocampus (SGZ, V‐SVZ) (Figure 2E), heart (Figure 2F), and duodenum (Figure 2G). The results showed that they were significantly increased in the D‐gal group compared to the Young group, which was mitigated by PTP, with a greater effect in the PTP/2w group than the PTP/1w group.

Fifth, given the key role of vascular endothelial dysfunction in aging, we also investigated the changes of NO, SA‐β‐gal, and aging‐related markers P16 and P21 within the aorta of rats. The fluorescence intensity of NO in aortas was decreased in the D‐gal group compared to the Young group, which was significantly recovered by PTP, with greater effect in the PTP/2w group than the PTP/1w group (Figure 2H). And SA‐β‐gal^+^ cells in the aorta of rats were significantly increased in the D‐gal group compared to the Young group, which was mitigated by PTP, with greater effect in the PTP/2w group than the PTP/1w group (Figure 2I). Similar results were found with regard to western blot of P16 and P21 in endothelial cells (Figure S2bI).

Sixth, we employed Oil red O staining on rat skeletal muscle and micro‐CT scanning for subsequent reconstruction analysis of femoral bone. The results exhibited a more obvious lipidosis in the D‐gal group compared to the Young group, which was significantly mitigated by PTP, with greater effect in the PTP/2w group than the PTP/1w group (Figure 2J). Murine femoral bone samples were collected for micro‐CT scanning (Figure 2K). Quantitative assessments showed a trend toward a decline in the bone volume fraction and trabecular number in the D‐gal group compared to the Young group, and these changes were partially attenuated by PTP (Figure 2L). Additionally, trabecular separation was elevated in the D‐gal group compared to the Young group, which was also somewhat attenuated by PTP (Figure 2L). Taken together, these results suggest that PTP may help mitigate certain aspects of skeletal aging, but further studies are needed to confirm these effects.

Finally, Hematoxylin and Eosin (HE) staining was employed to examine the morphological alterations and Masson's trichrome staining was utilized to assess the level and distribution of collagen in the murine skin (Figure 2M). Compared to the Young group, a significant reduction in the thickness of the epithelium and dermis was found in the D‐gal group, accompanied by a significant decrease in collagen content and a disordered collagen arrangement. Notably, PTP reversed these changes (Figure 2N). Compared with the young rats, the D‐gal group exhibited thicker glomerular capillary and more inflammatory cell infiltration in the kidney, and thinner lamina propria and mucous membrane layers disorganization in the duodenum, and interrupted bone trabecular and increasing trabecular spaces in the sternum and femur. These changes were significantly reversed by PTP, with greater effect in the PTP/2w group than the PTP/1w group (Figure S2bJ). To assess the proliferative capacity of murine skin cells, we performed Ki67 immunohistochemistry (Figure 2M). Ki67^+^ cells were significantly reduced in the D‐gal group compared to the Young group, which was significantly mitigated by PTP (Figure 2N).

Collectively, these findings indicate that PTP has the potential to rescue functional deficits in various organs and tissues in rats or mice and reverse the expression levels of aging‐related markers in multiple organs and tissues.

### 2.3 PTP Intervention Mitigates the Decline in Neurogenesis and Neuron Excitability Associated With Aging

Based on the above experiments, we conducted a more detailed investigation into the impact of PTP on neurogenesis and neural stem/progenitor cells in aged conditions.

**FIGURE 3:**
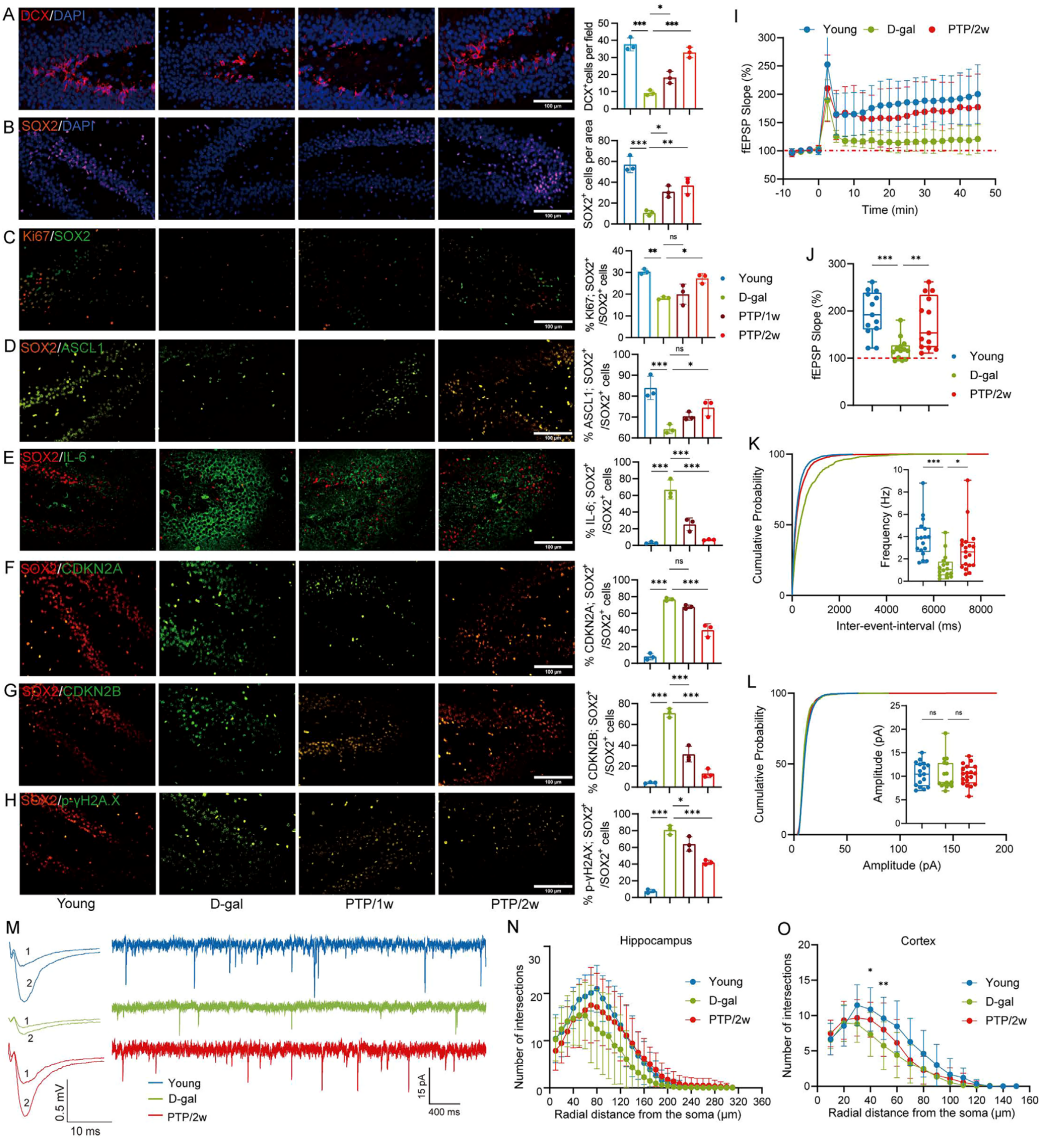
PTP intervention mitigates the decline in neurogenesis and excitability of neurons. (A) Representative immunofluorescence staining of DCX in hippocampus coronal DG of rats (scale bars, 100 μm), and quantification of DCX^+^ cells, *n* = 3/group. (B) Representative immunofluorescence staining of SOX2 in hippocampus coronal DG of rats (scale bars, 100 μm), and quantification of SOX2^+^ cells, *n* = 3/group. (C) Representative immunofluorescence staining of Ki67 and SOX2 in hippocampus coronal SGZ of rats (scale bars, 100 μm), and quantification of Ki67^+^SOX2^+^ cells, *n* = 3/group. (D) Representative immunofluorescence staining of ASCL1 and SOX2 in hippocampus coronal SGZ of rats (scale bars, 100 μm), and quantification of ASCL1^+^SOX2^+^ cells, *n* = 3/group. (E) Representative immunofluorescence staining of IL‐6 and SOX2 in hippocampus coronal SGZ of rats (scale bars, 100 μm), and quantification of IL‐6^+^SOX2^+^ cells, *n* = 3/group. (F) Representative immunofluorescence staining of CDKN2A and SOX2^+^ in hippocampus coronal SGZ of rats (scale bars, 100 μm), and quantification of CDKN2A^+^SOX2^+^ cells, *n* = 3/group. (G) Representative immunofluorescence staining of CDKN2B and SOX2 in hippocampus coronal SGZ of rats (scale bars, 100 μm), and quantification of CDKN2B^+^SOX2^+^ cells, *n* = 3/group. (H) Representative immunofluorescence staining of p‐γH2A.X and SOX2 in hippocampus coronal SGZ of rats (scale bars, 100 μm), and quantification of p‐γH2A.X^+^SOX2^+^ cells, *n* = 3/group. (I) Normalized fEPSP slope before and after the HFS protocol in the Young (blue), D‐gal (green), and PTP/2w (red) groups of mice. (J) The summary data measured 35–45 min after LTP induction (Young: 194.6% ± 46.94%, *n* = 13 slices from 4 mice; D‐gal: 119.6% ± 23.75%, *n* = 13 slices from 5 mice; PTP/2w: 175.1% ± 55.23%, *n* = 13 slices from 5 mice). (K, L) Cumulative distribution plots and summary of mEPSC amplitude and frequency in the Young (blue), D‐gal (green), and PTP/2w (red) groups of mice in hippocampal CA1 pyramidal cells (Frequency: 3.85 ± 1.8 Hz of Young, *n* = 17 slices from 4 mice; 1.34 ± 1.11 Hz of D‐gal, *n* = 16 slices from 5 mice; 2.84 ± 2.02 Hz of PTP/2w, *n* = 19 slices from 5 mice; Amplitude: 10.50 ± 2.49 pA of Young, *n* = 17 slices from 4 mice; 10.41 ± 3.26 of D‐gal, *n* = 16 slices from 5 mice; 10.28 ± 2.12 of PTP/2w, *n* = 19 slices from 5 mice). (M) Upper: Superimposed representative averaged fEPSCs recorded 10 min before (1) and 35–45 min after (2) LTP induction in the Young (blue), D‐gal (green), and PTP/2w (red) groups of mice. Below: Example traces of mEPSCs measured in CA1 hippocampal pyramidal cells from the Young (blue), D‐gal (green), and PTP/2w (red) groups of mice. (N) Number of intersections by radial distance from soma at hippocampus, 15 neurons from 3 samples. (O) Number of intersections by radial distance from soma at cortex, 15 neurons from 3 samples. Statistical analysis: One‐way ANOVA and Bonferroni post‐test for (I–L) and Tukey post‐test for the others. Data are mean ± SD; error bars denote 95% CI; *p < 0.05, **p < 0.01, ***p < 0.001.

Initially, we employed immunofluorescence staining to detect doublecortin (DCX) as the marker of immature neurons in the dentate gyrus (DG) of rats' hippocampus. A significant decline in DCX‐positive DG neurons was found in the D‐gal group compared to the Young group, which was significantly reversed in the PTP/2w group compared to the D‐gal group (Figure 3A). The results suggest a reduction in neurogenesis within this region. Consequently, we performed immunofluorescence staining on sections of the subgranular zone (SGZ) using the pan‐precursor marker SOX2 (Figure 3B) to label neural progenitor cells (NPCs) and the proliferation marker Ki67 (Figure 3C) to assess their proliferative status. The quantity of NPCs (SOX2^+^) in the SGZ was significantly reduced in the D‐gal group compared to the Young group. Additionally, the proportion of NPCs (SOX2^+^) expressing Ki67 was also diminished in the D‐gal group compared to the Young group. However, these changes were significantly reversed in the PTP/2w group (Figure 3B,C). These results suggest that both the quantity and proliferation of NPCs decline with aging, which could be restored through PTP.

Given that NPCs (SOX2^+^) in adult SGZ encompass both transit amplifying (TA) cells and stem cells, we conducted a separate characterization of these two populations. Immunofluorescence staining analysis revealed a significant reduction of TA cell marker ASCL1 in SOX2‐positive cells in the D‐gal group compared to the Young group, which was reversed in the PTP/2w group (Figure 3D). In contrast, the quantity of SOX2^+^ astrocytes did not exhibit significant changes between the D‐gal and Young groups, as evidenced by immunofluorescence staining for the combination of GFAP and SOX2 (Figure S3A). To validate these findings, we conducted an analysis of hippocampal sections for four additional markers indicative of cellular senescence. We found significantly elevated expression levels of IL‐6, CDKN2A, and CDKN2B, as well as the accumulation of DNA damage, evidenced by p‐γH2A.X (Figure 3E–H) in the D‐gal group compared to the Young group. These changes were significantly reversed in the PTP/2w group. These results suggest that an age‐related increase in the senescence of NPCs could be restored through PTP.

Given the memory improvements by PTP, we further analyzed synaptic connectivity in hippocampal slices of D‐galactose‐induced aged mice using electrophysiological recordings of LTP, fEPSPs, and mEPSCs. LTP experiments at the CA1 synapse were performed, fEPSPs and mEPSCs were measured before and after induction in hippocampal slices (Figure 3I–L). Consistent with improved spatial learning performance, LTP was also enhanced in D‐galactose‐induced aged mice undergoing PTP. In particular, PTP significantly mitigates aging‐related decline in the frequency of mEPSCs and shifted the cumulative probability curves of mEPSCs frequency to the left (Figure 3K), with no significant change in amplitude (Figure 3L). These quantitation experiments indicate that PTP partially reverses the aging‐related decline of synaptic plasticity, providing an electrophysiological basis for the improved memory performance.

Given that the changes of synaptic plasticity are associated with growth of neurites, morphology of neurons, and synaptogenesis, we performed Golgi stains and Sholl analysis (Figure S3B) to quantify neuronal complexities traced in cortical (Figure 3O) and hippocampal neurons (Figure 3N). Neuronal complexity in murine brain, manifesting as neurons' dendrite intersections in hippocampus, significantly decreased in the D‐gal group compared to the Young group. The decrease was significantly reversed in the PTP/2w group. There was no significant change in total dendritic length (Figure S3C,E) and the number of dendritic branches (Figure S3D,F) in both the basal and apical regions of the cortex and hippocampus among groups. Collectively, these experiments provide a morpho‐structural basis for the significant recovery of neural plasticity properties in D‐galactose‐induced aged animals observed in electrophysiological assessments after PTP.

We further analyzed the effects of PTP on the expression of proteins related to neuronal morphogenesis, including synaptophysin, postsynaptic density protein 95 (PSD95), and the N‐methyl‐D‐aspartate receptor subunit (NR2B), and analyzed the effects of PTP on the development of neuronal dendrites and dendritic spines and synaptic plasticity in combination with morphological changes. Our findings indicate that the expression levels of synaptophysin, PSD95, and NR2B were significantly decreased in the hippocampus of D‐galactose‐induced aged mice, compared with young mice. PTP produced a significant upregulation of PSD95, with similar trends with regard to synaptophysin and NR2B (Figure S3G).

In aging‐induced neurodegenerative diseases, NFL and tau often assess the full spectrum of neurologic senescence. We further utilized western blot analysis to examine tau‐5, p‐tau, and NFL in the cortex and hippocampus of rats (Figure S3H,I). The levels of p‐tau/tau‐5 and NFL significantly increased in D‐gal vs. Young group, which was significantly reversed following PTP. Given the pivotal role of Sirtuin 2 (Sirt2) in the processes of aging and cognitive decline, we also investigated Sirt2 in the cortex and hippocampus. The levels of Sirt2 significantly decreased in D‐gal vs. Young group, which was significantly prevented following PTP (Figure S3H,I). Collectively, these results suggest that PTP can effectively mitigate the decline in neurogenesis and excitability of neurons associated with aging, as well as the important markers of brain aging.

### 2.4 PTP Produces Anti‐Aging Through Restoring Function in Peripheral Blood and Immune Organs

To evaluate the effect of PTP on plasma from animals aged by the D‐gal treatment, we conducted an analysis of various aging‐related biomarkers in the blood.

**FIGURE 4:**
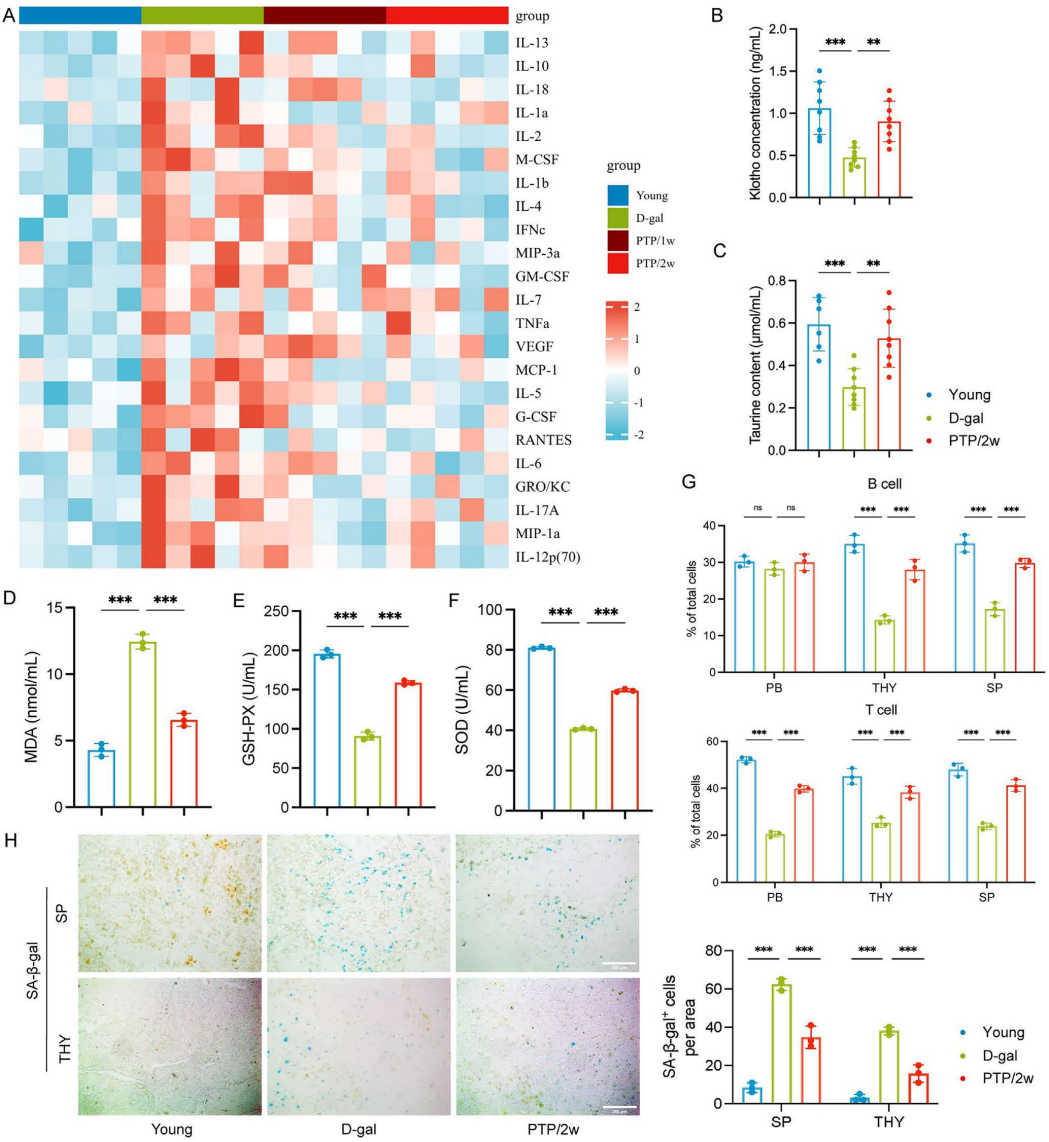
PTP produces the anti‐aging effect through the rejuvenation of peripheral blood. (A) Multiple cytokine profiling quantification of SASP proteins in rat peripheral blood plasma normalized to the D‐gal group, *n* = 5/group, using the Luminex multiplex assays. (B) Klotho concentration (ng/mL) in rat peripheral blood serum, *n* = 6–8/group. (C) Taurine content (μmol/mL) in rat peripheral blood serum, *n* = 6–8/group. (D) MDA (nmol/mL) in rat peripheral blood serum, *n* = 3/group. (E) GSH‐PX (U/mL) in rat peripheral blood serum, *n* = 3/group. (F) SOD (U/mL) in rat peripheral blood serum, *n* = 3/group. (G) B cell and T cell percentage of total cells from rat peripheral blood (PB), thymus (THY), and spleen (SP) were detected by flow cytometry, *n* = 3/group. (H) Representative SA‐β‐gal staining in spleen and thymus of mice (scale bars, 200 μm), and quantification of SA‐β‐gal^+^ cells, *n* = 3/group. Statistical analysis: One way ANOVA, Bonferroni post‐test for (J) and Tukey post‐test for the others. Data are mean ± SD; error bars denote 95% CI; *p < 0.05, **p < 0.01, ***p < 0.001.

First, we evaluated the levels of the senescence‐associated secretory phenotype (SASP) in the peripheral blood plasma of rats. The levels of SASP were significantly higher in the D‐gal group compared to the Young group, which were reversed in the PTP/2w group (Figure 4A). The same results were observed in the plasma of mice (Figure S4A). Second, given the key role of several serum biomarkers including the well‐known longevity protein klotho and taurine in aging, we investigated the effect of PTP on them. The results showed that these serum biomarkers were significantly lower in the D‐gal group compared to the Young group, which was also reversed in the PTP/2w group (Figure 4B,C). Third, given the key role of oxidative stress in aging, we quantified the levels of MDA, SOD, and GSH‐PX in peripheral blood. A significant increase of MDA and decrease of SOD and GSH‐PX were found in the D‐gal group compared to the Young group, and these changes were reversed in the PTP/2w group (Figure 4D–F). Finally, we quantified the levels of T cells (CD3^+^), and B cells (B220^+^) in peripheral blood and immune organs (thymus and spleen). Flow cytometry results indicated that T cells from peripheral blood, thymus, and spleen were significantly lower in the D‐gal group compared to the Young group, which was reversed in the PTP/2w group. In the spleen and thymus, we also observed the same changes in B cells, but there were no significant differences in peripheral blood (Figure 4G). Beyond this, we investigated the effect of PTP on the blood cell count of peripheral blood and the results indicated no significant difference among groups (Figure S4B–I). To further confirm the association of T cells and B cells in thymus and spleen with aging and the effect of PTP on them, we also observed the changes in β‐galactosidase (SA‐β‐gal) positive cells in thymus and spleen among groups. The quantity of SA‐β‐gal^+^ cells was significantly higher in the D‐gal group compared to the Young group, which were reversed in the PTP/2w group (Figure 4H).

These findings suggest that PTP can increase the levels of anti‐aging‐related factors in the blood plasma, significantly reverse the aging‐associated depletion of T cells in both peripheral blood and immune organs, and restore B cell populations within immune organs. This restoration of key lymphocyte populations (particularly T cells system‐wide and B cells within lymphoid tissues) contributes to an improved cellular foundation. By promoting this replenishment and enhancing anti‐aging factors, PTP acts to restore the systemic blood signaling environment to a more functional and healthy state. This restored circulatory milieu may subsequently create favorable conditions for the potential functional improvement of adaptive immunity and contribute to rescuing functional deficits in multiple tissues and organs.

### 2.5 PTP Promotes the Transformation of Hematopoiesis to a Youthful State by Improving the Hematopoietic Microenvironment in the Bone Marrow

To test the hypothesis that the restored functional and healthy state of peripheral blood after PTP is attributed to the mitigation of aging phenotypes in hematopoietic status of the bone marrow, a series of tests were conducted on the bone marrow.

**FIGURE 5:**
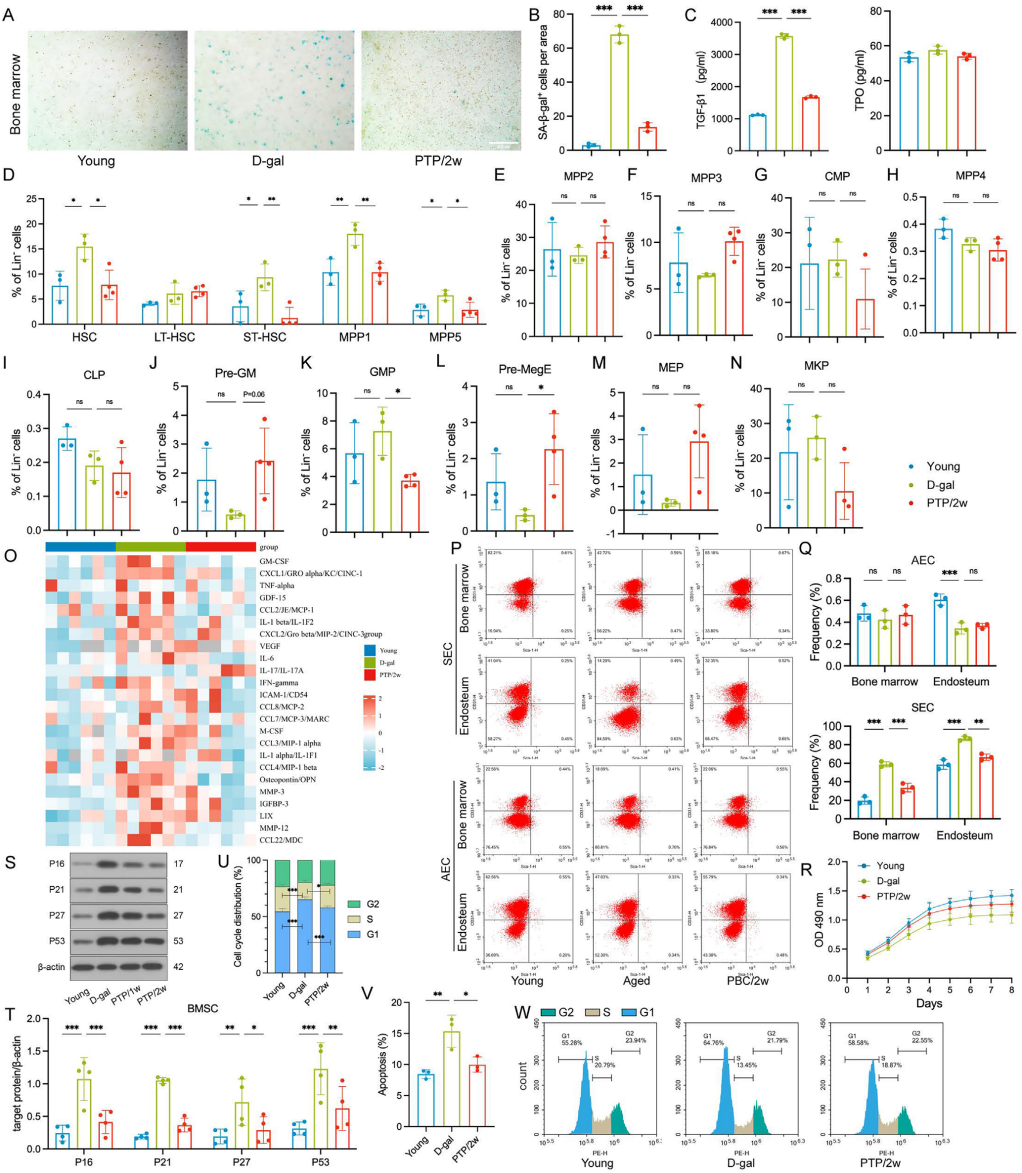
PTP promotes the transformation of hematopoiesis to a youthful state by improving the hematopoietic microenvironment in the bone marrow. (A) Representative SA‐β‐gal staining of rat bone marrow (scale bars, 200 μm) (B) Quantification of SA‐β‐gal^+^ cells per area, *n* = 3/group. (C) TGF‐β1 (pg/mL) and TPO (pg/mL) in rat bone marrow, *n* = 3/group. (D–N) The proportion of lin^−^ cell subsets (HSC, LT‐HSC, ST‐HSC, MPP1, MPP5, MPP2, MPP3, CMP, MPP4, CLP, Pre GM, GMP, Pre MegE, MEP, MKP) in mice bone marrow cells, *n* = 3–4/group. Statistical analysis: One‐way ANOVA and Bonferroni post‐test. (O) Multiple cytokine profiling quantification of SASP proteins in mice bone marrow, log2‐transformed fold change in mean fluorescence intensity (MFI) compared to the average of D‐gal group, *n* = 6/group. (P) Representative examples and (Q) quantification of SEC (CD31^low^Sca‐1^low^) and AEC (CD31^hi^Sca‐1^hi^) in ECs from rat bone marrow and endosteum was detected by flow cytometry, *n* = 3/group. (R) The third generation of rat bone marrow‐derived BMSCs were cultured for 8 days. Cell viability was measured by MTT assay, *n* = 5/group. (S–T) Western blot of P16, P21, P27, and P63 expression in rat bone marrow‐derived BMSCs, *n* = 4/group. (U) Cell cycle analysis of rat bone marrow‐derived BMSCs by flow cytometry, *n* = 3/group. (V) Quantification of cell cycle phase distribution, *n* = 3/group. (W) Apoptosis of the third generation of rat bone marrow‐derived BMSCs using Annexin V‐FITC/PI KIT combined with flow cytometry, *n* = 3/group. Statistical analysis: One‐way ANOVA, Bonferroni post‐test for (D–N) and Tukey post‐test for the others, Data are mean ± SD; error bars denote 95% CI; *p < 0.05, **p < 0.01, ***p < 0.001.

First, we determined the overall aging level of the bone marrow among groups through the changes in β‐galactosidase (SA‐β‐gal) positive cells in bone marrow. A significant increase was found in the D‐gal group compared to the Young group, which was reversed in the PTP/2w group (Figure 5A,B). Second, we measured the hematopoietic regulatory‐related factors TGF‐β1 and TPO (Figure 5C). The quantity of TGF‐β1 was significantly higher in the D‐gal group compared to the Young group, which was reversed in the PTP/2w group. But there was no discrepancy in TPO among the groups. Third, we performed a quantitative assessment of hematopoietic stem and progenitor cells within murine bone marrow utilizing flow cytometry. Compared to the Young group, there was an upregulation of hematopoietic stem cells (HSC), short‐term hematopoietic stem cells (ST‐HSC), and early multipotent progenitor cells (MPP1, MPP5) in the D‐gal group vs. Young group, which was significantly reversed by PTP treatment. However, long‐term hematopoietic stem cells (LT‐HSC) exhibited no notable changes among groups (Figure 5D). These results suggest that upstream hematopoietic stem cells and early multipotent progenitor cells tend to remain in a quiescent state with respect to proliferation and differentiation under aging conditions, but they can be activated by PTP. No significant differences were observed for biased‐multipotent progenitor cells (MPP2, MPP3, and MPP4), common myeloid progenitor cells (CMP), and common lymphoid progenitor cells (CLP) among groups (Figure 5E–I). In the D‐gal group, the levels of pre‐granulocyte‐macrophage progenitors (Pre‐GM) declined, which was also restored by PTP (Figure 5J). Conversely, granulocyte‐macrophage progenitor (GMP) levels were significantly elevated in the D‐gal group, which was significantly prevented by PTP (Figure 5K). Similarly, a significant reduction in pre‐megakaryocytic‐erythroid progenitors (Pre‐MegE) and megakaryocyte erythroid progenitor cells (MEP) was found in the D‐gal group, which was restored by PTP (Figure 5L,M). Conversely, a significant increase in megakaryocyte progenitor cells (MKP) was observed in the D‐gal group, which was reversed by PTP (Figure 5N). Bone marrow‐derived T cells and B cells declined in the D‐gal group compared to the Young group, which was reversed by PTP (Figure S5A). Comprehensively considering the above results, it is evident that myeloid hematopoiesis increased while lymphoid hematopoiesis decreased in the D‐gal group, which was consistent with the peripheral immune aging state. However, PTP significantly reversed this age‐associated hematopoietic alteration. The gating strategy of flow cytometry is shown (Figure S5B,C).

Additionally, we found a significant increase of SASP levels in the bone marrow (Figure 5O), which are analogous to those in the peripheral blood and various organs. Given that the bone marrow niche, which includes endothelial cells (ECs) and bone marrow mesenchymal stem cells (BMSCs), generates regulatory signals that are crucial for the modulation of hematopoietic function, a quantitative analysis of ECs derived from bone marrow and endosteum was performed using flow cytometry. In the D‐gal group, the quantity of sinusoidal ECs (SECs) significantly increased, and intervention with PTP was able to reverse this change (Figure 5P,Q). However, arterial ECs (AECs) were not affected by aging or PTP (Figure 5P,Q). To investigate the effects of aging and PTP on bone marrow mesenchymal stem cells (BMSCs), we cultured BMSCs from the rats and assessed the expression of several aging‐related markers (P16, P21, P27, P53). The results showed a significant increase in these aging markers in cultured BMSCs from the D‐gal vs. Young group, which was reversed in cultured BMSCs from PTP group (Figure 5S,T). The 3‐[4,5‐dimethylthiazol‐2‐yl]‐2,5‐diphenyl tetrazolium bromide (MTT) assay was used to measure the proliferation rate of bone marrow mesenchymal stem cells (BMSCs). The results showed that a significantly lower proliferation rate was observed in the D‐gal vs. the Young group, which was significantly reversed by PTP (Figure 5R). In the D‐gal group, the proportion of quiescent cells (G1 phase) was significantly higher compared to the Young group, while the percentage of cells in the S phase was notably lower in the D‐gal group relative to the Young group (Figure 5U,W). Furthermore, anti‐apoptosis experiments demonstrated that the proportion of apoptotic BMSCs in the D‐gal group was significantly greater than that observed in the Young group. Notably, all these aging‐related changes could be significantly reversed by PTP treatment (Figure 5V).

Collectively, our findings demonstrate that PTP can maintain the youthful functionality of bone marrow, as evidenced by its promotion of lymphoid‐biased proliferation and restoration of a balanced state of hematopoiesis. This, in turn, contributes to mitigating the aging process in various tissues and organs throughout the body.

## 3 Discussion

Current anti‐aging strategies, including senescent cell removal, stem cell therapy, and organ transplantation, are closely linked to anti‐inflammatory mechanisms (Li et al. 2023). Indeed, chronic inflammation accelerates the senescence of immune cells, leading to weakened immune function and an inability to clear senescent cells and inflammatory factors, creating a vicious cycle of inflammation and senescence (López‐Otín et al. 2023; Mogilenko et al. 2022). In addition, heterochronic parabiosis by blood exchange is also a promising anti‐aging strategy, which was also related to the restoration of anti‐inflammatory factors by young blood or plasma. However, the blood or plasma source is a big obstacle for translating into clinical practice. Our study provides preliminary evidence for the anti‐aging effect of PTP, which may drive peripheral blood functional restoration by reducing senescence markers in bone marrow, evidenced by decreased senescence‐associated phenotype levels in both circulation and tissues, thereby promoting systemic rejuvenation of both structural and functional aspects.

Our findings demonstrate that PTP reverses senescence‐associated structural deterioration and molecular alterations in the liver, kidney, skeletal muscle, bone, aorta, skin, and particularly the brain, including the expression levels of aging‐related markers in multiple organs and tissues. In addition, PTP mitigates the aging‐related decline in neurogenesis and neural excitability, cognition and motor performance. Thus, these results prove the anti‐aging effect of PTP. Furthermore, PTP is found to significantly decrease SASP levels and elevate plasma levels of the renowned anti‐aging factors, klotho and taurine, known for their roles in promoting longevity. This suggests that the anti‐aging effect of PTP may be mediated by peripheral blood function and reducing senescence markers. In agreement with the current results, a growing body of work has demonstrated that ‘young blood’ or a rejuvenated systemic environment can produce anti‐aging effect. For example, heterochronic parabiosis has been shown to rejuvenate aged muscle, liver, heart, pancreas, bone, spinal cord and brain, ultimately leading to an extension of life and healthspan (Iram et al. 2022; Conboy et al. 2005; Villeda et al. 2014; Lagunas‐Rangel 2024; Zhang et al. 2023; Tyshkovskiy et al. 2023; Pálovics et al. 2022). Various studies have demonstrated that systemic infusion of the plasma fraction from young blood is efficacious in reversing cognitive decline and regenerative impairments associated with the hippocampus (Villeda et al. 2014; Middeldorp et al. 2016). Taken together, these results suggest that PTP may share common anti‐aging mechanisms with heterochronic parabiosis, or systemic infusion of young blood, because these strategies will replenish aged blood. These blood‐based rejuvenation strategies underscore the significance of both introducing pro‐youth factors and inhibiting or reducing pro‐aging factors as complementary therapeutic avenues to counteract age‐related cognitive decline. Notably, regarding protocol optimization, the regimen of once every 2 weeks yielded superior efficacy compared to weekly treatment. We attribute this to the balance between hematopoietic stimulation and recovery. Since hematopoietic homeostasis typically requires approximately 1 week to restore (Diehl et al. 2001), weekly treatment may impose continuous stress. In contrast, the two‐week interval likely functions as an intermittent pre‐conditioning hormesis, allowing sufficient recovery to optimize regeneration without exhausting the stem cell pool (Kamota et al. 2009; Yu et al. 2016).

In bone marrow, hematopoietic stem cells (HSCs) maintain a balanced production of blood cells throughout life. With aging, the proportion of myeloid‐biased HSCs (My‐HSCs) increases relative to balanced HSCs (Bal‐HSCs) (Ross et al. 2024; Mitchell et al. 2023). Chronic low‐grade aging‐related inflammation directly contributes to the loss of endosteal mesenchymal populations, impaired osteogenesis, and vascular dysfunction (Poulos et al. 2017). These changes, along with the expansion of inflammatory mesenchymal stem cells, drive lineage biases and regenerative defects in the aged blood system (Frisch et al. 2019; Cain et al. 2025). Furthermore, inducing or restoring the youthful state of bone marrow was found to produce an anti‐aging effect. For example, heterochronic bone marrow transplantation, which replenished aged blood with young immune cells, enhanced synaptic density, reduced microglial activation, and improved hippocampal cognition in aged mice (Das et al. 2019). My‐HSCs depletion in aged mice allows Bal‐HSCs to rebalance the hematopoietic system, restoring youthful immune characteristics (Ross et al. 2024; Kovtonyuk et al. 2022). This rejuvenation is marked by increased lymphocyte progenitors and naive cells, reduced markers of lymphocyte dysfunction and exhaustion, and lower levels of inflammatory mediators. Given the key role of bone marrow in aging and the possible effect of PTP on the mobilization and recruitment of bone marrow stem cells (Liu et al. 2024; Montecino‐Rodriguez et al. 2019), we hypothesize that the anti‐aging effect of PTP through restoring peripheral blood function should be attributed to its effect on bone marrow rejuvenation. Consistent with this hypothesis, we find that PTP promotes the rejuvenation of the bone marrow niche, which includes ECs and BMSCs. Specifically, PTP treatment in D‐galactose‐treated mice and rats led to a favorable alteration in the proportion of sinusoidal ECs (SECs) and arterial ECs (AECs) within the bone marrow and endosteum. Moreover, PTP enhanced the functional state of BMSCs, as demonstrated by the restoration of proliferative capacity and a reduction in the expression of senescence‐associated factor levels. This process subsequently promotes lymphocyte‐biased proliferation of hematopoietic cells and the periodic clearance of SASP. This potentially benevolent cycle maintains the youthful function of the bone marrow and rebuilds the hematologic system, suggesting a potential role in the anti‐aging effect of PTP. However, to confirm the mechanistic links between systemic changes in blood composition and the observed tissue improvements, further experiments are required, particularly to investigate how perturbation of blood functional restoration may impact the downstream rejuvenating effects on the bone marrow and other tissues. These studies will be essential to establish the causality of the observed effects.

The main strength of this study is the first report demonstrating that periodic therapeutic phlebotomy produces anti‐aging effects by mitigating aging phenotypes in the bone marrow microenvironment and reducing senescence markers, subsequently restoring peripheral blood function and reducing systemic senescence‐associated secretory phenotypes. This easy and safe anti‐aging strategy would overcome the dilemma of limited sources of plasma exchange and help to solve the difficulties of clinical blood scarcity, achieving the dual role of “helping others as well as benefiting ourselves”. A limitation of our study is the lack of testing the effect of PTP in female rats, although mice studies did not reveal any sex‐based differences in certain outcomes. Second, we utilized the D‐galactose‐induced aging model, which was chosen for its convenience, minimal side effects, and higher survival rate (Azman and Zakaria 2019; Gao et al. 2021). However, it is important to acknowledge that while the D‐gal model is widely used, it does not fully replicate the complexity of natural aging, which involves a broader range of factors, including genetic variability, epigenetic changes, and multi‐organ system decline. The natural aging model remains the most reliable and comprehensive approach for investigating the complexities of aging. To address this limitation, we are conducting a separate study to investigate the effects of PTP in naturally aging mice, which will provide a more robust understanding of how PTP treatment influences aging in a more physiologically relevant context. Third, given the effect of human heterogeneity in iron metabolism and genetic backgrounds (Rosenblum 2023; Zeidan et al. 2021), dysregulated iron metabolism plays a significant role in aging and age‐related diseases, particularly in cardiovascular and neurological functions. These factors highlight the importance of considering iron homeostasis when evaluating the therapeutic outcomes of PTP treatment, which involves blood rejuvenation. Therefore, this finding underscores the need for clinical trials that account for the variability in iron metabolism and genetic factors to accurately assess the effects of PTP across diverse populations. Fourth, while this study suggests a role for BMSC and endothelial cells in rejuvenation, the findings remain correlative and lack functional validation. Previous studies have highlighted the role of the bone marrow microenvironment in aging and rejuvenation, providing important context for our findings (Kuribayashi et al. 2021; Mitchell et al. 2023; Ambrosi et al. 2021; Ho et al. 2021). Future research should include functional assays, such as bone marrow transplantation, to confirm these mechanisms. Additionally, while BMSC proliferation and blood vessel formation were quantified, further studies are needed for functional validation. Fifth, this study did not evaluate the effects of PTP on untreated young healthy controls. We hypothesize that PTP would likely yield minimal observable benefits in young animals due to a physiological “ceiling effect,” as they inherently possess optimal hematopoietic function and low baseline inflammation. Finally, although we have developed a safe protocol of PTP based on systemic blood volume and recovery following blood loss, further investigation is necessary to optimize the PTP intervention strategy.

In conclusion, this preclinical study provides preliminary evidence suggesting that PTP exerts anti‐aging effects by reducing senescence markers in bone marrow and mitigating aging phenotypes, subsequently restoring peripheral blood function and reducing systemic senescence‐associated secretory phenotypes. Given its technical simplicity and safety profile, PTP holds potential as a clinically translatable strategy for age‐related disease prevention and management, offering new perspectives for developing practical anti‐aging therapies. However, further validation in a naturally aged model is necessary to confirm these findings.

## 4 Materials and Methods

### 4.1 Animals and Drug Treatments

Eight‐week‐old male Sprague–Dawley rats and male and female C57BL/6J mice were obtained from the Experimental Animal Center, Hospital of Northern Theater Command, China. Both male and female mice were used in equal numbers in the experiments. We established D‐galactose‐induced aging model by subcutaneously injecting 150 mg/kg of D‐galactose (Absin, abs816779) into rats or mice daily for 8 weeks. The Young group received placebo injections, which consisted of an equal volume of physiological saline (0.9% NaCl), as a vehicle control. At the four‐week following the initiation of injections, the D‐gal‐injected rats were divided into three subgroups: the D‐gal group (no bloodletting), the PTP/1w group (weekly bloodletting), and the PTP/2w group (once every 2 weeks bloodletting). The D‐gal‐injected mice were divided into two subgroups: the D‐gal group (no bloodletting) and the PTP/2w group (once every 2 weeks bloodletting). All experiments were approved by the Institutional Animal Care and Use Committee of General Hospital of Northern Theater Command (Ethics Number: 2020008) and conformed to the National Institutes of Health Guide for the Care and Use of Laboratory Animals.

### 4.2 Periodic Therapeutic Phlebotomy and Blood Sample Collection

The PTP intervention was conducted as established frequency, weekly bloodletting and once every 2 weeks bloodletting, respectively. During bloodletting, the neck of the rats and mice were gently but firmly grasped to ensure proper support without causing asphyxiation. For blood collection, the thumb and forefinger of the left hand were used to gently press the sides of the neck of the rats or mice, partially obstructing venous return to the head, resulting in protrusion of the eyeballs and congestion of the orbital venous plexus. While holding a long‐necked (3–4 cm) rigid capillary glass tube with an inner diameter of 0.5 to 1.0 mm, the tip was inserted into the orbital venous plexus by piercing the inner angle between the eyelid and the eyeball at a 45° angle toward the lacrimal gland region, and the capillary glass tube was rotated to incise the arterial sinus, where the blood flowed into the centrifuge tube. Once a prespecified amount of blood had been collected, pressure on the neck was released and the blood collection device was removed. To control for procedural stress while avoiding the risk of unintended hemorrhage associated with orbital pressure, mice in the Young and D‐gal groups were subjected to comparable handling and physical restraint sessions at the same frequency as the PTP group. Given that the average circulating blood volumes of rats and mice are approximately 64 mL/kg and 72 mL/kg, respectively, and that homeostasis can be restored within 1 week when a single blood collection does not exceed 7.5% of the total circulating blood volume (Diehl et al. 2001). Therefore, the standard bloodletting procedure is to draw 6% of their total circulating blood volume calculated based on their body weight. To prevent anemia, replace lost fluids with an equal amount of saline intraperitoneally.

### 4.3 Tissue Isolation

For histological examination, tissues were harvested postmortem. Specimens designated for the SA‐β‐galactosidase assay were promptly cryopreserved in liquid nitrogen and subsequently embedded with the Tissue‐Tek O.C.T. compound kit (Sakura, 4583). Samples intended for the Oil Red O assay underwent initial fixation in formaldehyde, followed by dehydration in 20% and 30% sucrose solutions, and were ultimately embedded using the identical Tissue‐Tek O.C.T. compound kit. Additional experimental procedures utilized paraffin embedding methodologies.

### 4.4 Morris Water Maze

The Morris water maze setup includes a circular pool, an underwater platform, and an automated imaging system with a camera and analysis software. In this study, the pool is 150 cm in diameter and 40 cm high, with a dark interior and water at 24 cm depth and (21 ± 1)°C. Lighting is controlled to avoid direct illumination. The pool is divided into four quadrants, with a 12 cm diameter platform in quadrant 1, placed 20 cm from the pool wall and submerged 1 cm below the surface. An overhead camera records footage for analysis.

### 4.5 Experimental Design and Platform Quadrant Configuration

The study uses the Morris water‐maze test over 6 days, with the first five for positioning navigation and the last for spatial exploration. The platform is in quadrant 1, opposite quadrant 3.

### 4.6 Positioning Navigation Test

The rat is placed in the pool from each of the four quadrants for up to 90 s. If it fails to find the platform, it is guided there and stays for 15 s before being dried and returned to its cage. This is done daily from each quadrant for 5 days. The average time taken to reach the platform from all quadrants measures spatial learning ability.

### 4.7 Spatial Exploration Test

On day six of the experiment, the environmental conditions and water temperature remained consistent with the positioning navigation test. The submerged platform was removed, and a rat was placed in quadrant 3 of the pool. Its swimming path was recorded for 90 s and analyzed to evaluate spatial memory. The analysis focused on how often the rat crossed the former platform's location and the percentage of time spent in quadrant 1, the target area.

### 4.8 Y‐Maze

The Y‐maze, with three arms each 45 cm long, 9 cm wide, and 27 cm high, is arranged at equal angles. Rats and mice started at one arm's end and explored freely for 6 min. An arm entry was noted when the animal's hind paws were fully inside an arm. Spontaneous alternation was the exploration of all three arms without immediate repetition. The alternation percentage was calculated by dividing the number of alternations by the total arm entries minus two, then multiplying by 100%.

### 4.9 Open Field Test

The Open field test involved placing rats in a 100 cm square arena and mice in a 50 cm square arena, both with gray interiors and no visual cues. After a 24‐h acclimatization, each animal was placed at the center for a five‐minute exploration session. A video tracking system recorded metrics like total distance traveled and time spent in the central 25% of the arena.

### 4.10 Treadmill

#### 4.10.1 Running‐Table Experiment

Mice were acclimated to a treadmill over 2 days. On day one, they walked at 6 cm/s for 2 min. Day two mimicked the formal test but lasted 2 min, training mice to avoid the shock grid, starting at 10 cm/s with gradual speed increases. On day three, formal testing began at 6 cm/s, increasing by 1 cm/s every 20 s, measuring endurance until the mice touched the shock grid three times.

### 4.11 Forced Swim Test (FST)

The circular swimming tank, 18 cm in diameter and 50 cm high, was filled with water to a depth of 30 cm at a temperature of 21°C ± 1°C. Mice, with a weight attached to their tails equal to 5% of their body weight, swam freely. The exhaustion latency of the mice was defined as the time interval from when their heads remained submerged in the water for more than 5 s until they ceased to resurface.

### 4.12 Tail Suspension Test (TST)

The mice were placed on a 2 mm metal wire, gripping it with all limbs. Hanging endurance was measured by multiplying hanging time (seconds) by body weight (grams), averaged over three trials per mouse.

### 4.13 SA‐β‐Galactosidase Staining

The Senescence Cells Histological Staining Kit (Beyotime, C0602) was used for SA‐β‐gal staining according to the manufacturer's instructions. Senescent cells appeared blue under a light microscope (OLYMPUS, BX53). The number of SA‐β‐gal‐positive cells per unit area was quantified by analyzing three random image fields using Image J's (ij154‐win‐java8, National Institutes of Health, Bethesda, USA) color‐threshold function.

### 4.14 Histological Analysis

Tissue sections from rats and mice were prepared using standard methods. Hematoxylin–eosin, Masson's trichrome, Oil Red O, and Sirius Red stains were performed according to standard procedures. Collagen fiber positivity was quantified with Image‐Pro Plus software, and the percentage was calculated as: (collagen‐stained area/total dermal area) × 100%.

### 4.15 Multiplex Protein Analysis Using Luminex

Cytokine and signaling molecule levels were measured using standard antibody‐based multiplex immunoassays (Luminex rat 23‐plex and mouse 24‐plex immunoassays). Mean fluorescence intensity values were used for analysis, and all measurements were blinded. The heatmap was generated utilizing the ComplexHeatmap package, employing a Z‐score transformed data matrix with row normalization. The analysis was conducted using R software version 4.2.1 and the ComplexHeatmap package version 2.13.1.

### 4.16 Enzyme‐Linked Immunosorbent Assays

The concentration of Klotho (Elabscience, E‐EL‐R2580c), Taurine (Absin, abs580222), TGF‐β1 (Multi Sciences, EK981), TPO (Fine Test, ER0202) was measured in accordance with the protocols outlined in the respective reagent kit manuals.

### 4.17 Blood Analysis

For the blood routine examination, 50 μL of fresh blood was collected from each rat and mouse and immediately mixed with EDTA. Routine blood test was performed using BC‐2800 Vet Automatic animal blood cell analyzer (Mindray). For serum biochemical analysis, blood samples were allowed to clot for 2 h at room temperature, followed by centrifugation at 1000 *g* for 10 min to obtain serum. An aliquot of 200 μL of serum was then analyzed for total bilirubin (TBIL), blood urea nitrogen (BUN), creatinine, malondialdehyde (MDA), alanine aminotransferase (ALT), superoxide dismutase (SOD), and glutathione peroxidase (GSH‐Px) using the Chemray‐800 chemistry analyzer (Rayto), in accordance with the manufacturer's recommended reagents and settings.

### 4.18 Immunohistochemistry

The skeletal muscles, kidneys, and liver were embedded in paraffin, and tissue sections measuring 5 μm were subsequently obtained. For immunohistochemical (IHC) analyses, the deparaffinized and rehydrated sections underwent heat‐induced epitope retrieval by incubation in 1 mM citrate buffer (pH 6.0) at 95°C for 30 min using a microwave oven. Following this, the sections were treated with 3% hydrogen peroxide for 20 min to inhibit endogenous peroxidase activity. To prevent non‐specific binding, the sections were incubated with 1% bovine serum albumin (BSA) for 15 min at room temperature (25°C). Primary antibodies against p‐γH2A.X (Abclonal, AP0687), HMGB1 (Wanleibio, WL03023), Ki‐67 (Abcam, ab15580), and Desmin (Wanleibio, WL0174) were then applied overnight at 4°C. After washing with phosphate‐buffered saline (PBS), the sections were incubated with a secondary antibody, Goat anti‐Rabbit IgG (H + L) Secondary Antibody (Thermo Fisher Scientific Inc., catalog number: 31460), for 1 h at 37°C. The images obtained from the immunohistochemical experiments were analyzed using ImageJ (ij154‐win‐java8, National Institutes of Health, Bethesda, USA).

### 4.19 Immunofluorescence

Tissue sections, each measuring 5 μm in thickness, underwent deparaffinization and rehydration processes, followed by antigen retrieval using a citrate buffer. The sections were subsequently blocked with bovine serum albumin (BSA) for 15 min and rinsed three times with phosphate‐buffered saline (PBS). The slides were then incubated overnight at 4°C with primary antibodies, specifically targeting DCX (RD, ab207175), SOX‐2 (RD, AF2018), Ki67 (Novus, NBP2‐22112SS), ACSL1 (Affinity, DF8504), GFAP (Affinity, DF6040), p‐γH2A.X (Abclonal, AP0687), CDKN2A (Santa Cruz, Sc‐1661), and CDKN2B (Invitrogen, PA5‐49749) and IL‐6 (Abclonal A21264). Subsequently, the slides were treated with a Cy3 or Alexa Fluor 488‐conjugated secondary antibody. Following three additional washes, the nuclei were counterstained with DAPI. The mounted sections were then examined and imaged using fluorescence microscopy.

### 4.20 NO Imaging of Living Endothelial Cells

NO Imaging of Living Endothelial Cells NO Imaging was performed as described previously (Azman and Zakaria 2019). In short, slices were loaded with 20 μM diamino‐fluorescein‐2‐diacetate (DAF‐2DA; Glpbio Technology Inc., China) for 30 min at 37°C and quantified by staining with background fluorescence using epifluorescence.

### 4.21 Electrophysiology

Mice were anesthetized with isoflurane, following which their brains were swiftly extracted and immersed in an ice‐cold solution comprising 235 mM sucrose, 1.25 mM NaH_2_PO_4_, 2.5 mM KCl, 0.5 mM CaCl_2_, 7 mM MgCl_2_, 20 mM glucose, 26 mM NaHCO_3_, and 5 mM pyruvate (pH 7.3, 310 mOsm, saturated with 95% O_2_ and 5% CO_2_). Sagittal hippocampal slices (350 μm) were prepared utilizing a vibrating slicer (Leica, VT1200) and subsequently incubated for 30 to 40 min in artificial cerebrospinal fluid (ACSF). The composition of the ACSF included: 26 mM NaHCO_3_, 3.5 mM KCl, 126 mM NaCl, 10 mM D‐glucose, 1 mM sodium pyruvate, 1.25 mM NaH_2_PO_4_, 2 mM CaCl_2_, and 1 mM MgCl_2_ (pH 7.4, 310 mOsm), and was also saturated with 95% O_2_ and 5% CO_2_.

The tissue slices were transferred to a submerged chamber and continuously perfused with oxygen‐saturated artificial cerebrospinal fluid (ACSF) at a flow rate of 3 mL/min. To facilitate the monitoring of miniature excitatory postsynaptic current (mEPSC) activity in CA1 pyramidal neurons, tetrodotoxin (TTX, 1 μM) and picrotoxin (100 μM) were incorporated into the ACSF to inhibit network activity and block GABAergic receptors, respectively. The pipettes used for voltage‐clamp recordings in granule cells contained the following components: 125 mM CsMeSO_3_, 10 mM HEPES, 10 mM EGTA, 8 mM TEA‐Cl, 5 mM 4‐AP, 0.4 mM GTP‐Na, 4 mM ATP‐Na2, 1 mM CaCl_2_, and 1 mM MgCl_2_, with a pH of 7.3–7.4 and an osmolarity of 280–290 mOsm. The cells were voltage‐clamped at −70 mV and allowed to stabilize for at least 5 min prior to the initiation of recordings.

Electrical stimuli were delivered via a bipolar electrode (FHC) strategically placed in the Schaffer Collateral of the stratum radiatum, maintaining a 30‐s inter‐trial interval during both the baseline phase and subsequent to the induction of long‐term potentiation (LTP). The stimulus intensity was typically set to approximately 40%–50% of the threshold required to evoke population spikes. Throughout the experimental protocol, the stimulation intensity was consistently maintained within the range of 50 to 200 μA. Field excitatory postsynaptic potentials (fEPSPs) were recorded in current‐clamp mode using a glass pipette filled with artificial cerebrospinal fluid (ACSF). Following the establishment of a stable 10‐min baseline period, LTP was induced by administering three trains of high‐frequency stimulation (HFS), each comprising 100 pulses at a frequency of 100 Hz, with inter‐train intervals of 20 s.

Patch‐clamp recordings were conducted using Axopatch 700B amplifiers (Molecular Devices). The data collected were filtered at 6 kHz and sampled at 20 kHz, with subsequent off‐line processing performed using pClampfit 10.6 software (Molecular Devices). To assess series and membrane resistances, negative pulses of −10 mV were applied. Data were included in the analysis only if the series resistances demonstrated less than a 20% variation over the course of the trial. All experimental procedures were carried out at a controlled temperature of 32°C.

### 4.22 Golgi Staining

Mice were subjected to deep anesthesia, after which the entire brain was excised and incubated in Golgi‐Cox solution. This solution consisted of a mixture containing 5% K_2_Cr_2_O_7_, 5% HgCl_2_, and 5% K_2_CrO_4_, each dissolved in distilled water. The brains were maintained at room temperature for a period of 4 days. Subsequently, coronal sections of the hippocampus and cortex, each with a thickness of 150 μm, were prepared using a vibratome. The sections underwent a series of processing steps: initially, they were rinsed twice with distilled water for 5 min each. Following this, they were dehydrated in 50% ethanol for 5 min and incubated in an ammonia solution (composed of three parts ammonia to one part distilled water) for 10 min. This procedure was followed by two additional rinses in distilled water, each lasting 5 min, and a 10 min incubation in 5% sodium thiosulfate in the dark. Upon completing the rinsing, dehydration, and clearing procedures, the sections were imaged using the Pannoramic MIDI II system (3DHISTECH). Subsequent analyses were conducted utilizing Image J software. The apical and basal dendrites of individual pyramidal neurons were traced with the Neuroanatomy plugin in ImageJ, and Sholl analysis was performed using the Sholl Analysis SNT 4.2.1 plugin in ImageJ. Cortical images were captured at 63× magnification, while hippocampal slices were imaged at 41× or 36× magnification. Neurons were sampled from layers II/III of the cortex (15 per group) and the hippocampal CA1 region (15 per group). All images were standardized to a uniform scale, with measurements expressed in micrometers (μm). The dendritic arbors of Golgi‐impregnated pyramidal neurons, including both apical and basal dendrites in the hippocampal CA1 region and layers II/III of the cortex, were manually traced using the Simple Neurite Tracer (SNT) function in Image J. Tracing commenced from branches directly originating from the soma and proceeded centrifugally along each parent branch and its subsequent daughter branches. The evaluated dendritic characteristics included total length and branch number, and Sholl analysis, the latter of which quantified dendritic intersections at radial intervals of 10 μm.

### 4.23 Flow Cytometry of Hematopoietic Cells and Niche Cells

Phenotypic analysis of lineage cells, hematopoietic progenitor cells, and stem cells was conducted. The identification of distinct cell populations was facilitated through the use of specific cell surface markers, including LT‐HSC (Lin^−^Sca‐1^+^c‐Kit^+^CD34^−^CD48^−^CD135^−^), ST‐HSC (Lin^−^Sca‐1^+^c‐Kit^+^CD34^+^CD48^−^CD135^−^), MPP1 (Lin^−^Sca‐1^+^c‐Kit^+^CD150^+^CD48^−^CD34^+^CD135^−^), MPP2 (Lin^−^Sca‐1^+^c‐Kit^+^CD150^+^CD48^+^CD135^−^), MPP3 (Lin^−^Sca‐1^+^c‐Kit^+^CD150^−^CD48^+^CD135^−^), MPP4 (Lin^−^Sca‐1^+^c‐Kit^+^CD150^−^CD48^+^CD135^+^), MPP5 (Lin^−^Sca‐1^+^c‐Kit^+^CD150^−^CD48^−^CD135^−^), CMP (Lin^−^Sca‐1^−^c‐Kit^+^CD34^+^CD16/32^−^), GMP (Lin^−^Sca‐1^−^c‐Kit^+^CD34^+^CD16/32^+^), MEP (Lin^−^Sca‐1^−^c‐Kit^+^CD34^−^CD16/32^−^), MKP (Lin^−^Sca‐1^−^c‐Kit^+^CD150^+^CD41^+^), Pre GM (Lin^−^Sca‐1^−^c‐Kit^+^CD150^−^CD105^−^CD41^−^CD16/32^−^), Pre MegE (Lin^−^Sca‐1^−^c‐Kit^+^CD150^+^CD105^−^CD41^−^CD16/32^−^), CLP (Lin^−^Sca‐1^low^c‐Kit^low^CD135^+^CD127^+^). To detect T cells (CD3e) and B cells (B220), samples were stained with CD3e‐FITC and B220‐PE‐CY7. To detect CD31^low^Sca‐1^low^ sinusoidal EC (SEC) and CD31^hi^Sca‐1^hi^ arteriolar ECs (AECs) both in the central marrow and at the endosteum. Flow cytometric data were analyzed utilizing FlowJo and Cytexpert software.

### 4.24 Western Blotting

Tissue samples were extracted using a Whole Cell Lysis Assay kit (Wanleibio, WLA019). Protein concentrations were quantified with a BCA kit (Wanleibio, WLA004). Proteins were subsequently separated via SDS‐PAGE (Wanleibio, WLA013) and transferred onto polyvinylidine difluoride membranes. The membranes were blocked for 1 h using a solution of 5% dry milk in 0.15% Tween‐20 in TBS (TBST buffer). After blocking, membranes were washed and incubated with primary antibodies at 4°C overnight. Secondary antibodies were then applied and incubated at room temperature for 2 h. Following four washes with TBST buffer, immunoreactive bands were visualized using chemiluminescent detection (ECL). The optical density of the target bands was analyzed using Gel‐Pro Analyzer software. The primary antibodies used for Western blotting included: p53 (Wanleibio, WL01919), p27 (Wanleibio, WL04174), p21 (Wanleibio, WL0362), p16 (Wanleibio, WL01418), p‐tau (Abclonal, AP1096), tau‐5 (Affinity, AF6141), NEFL (Proteintech, 12998‐1‐AP), SIRT2 (Proteintech, 19655‐1‐AP), GH1 (Proteintech, 55243‐1‐AP), IGF (Abclonal, A11985), and β‐actin (Wanleibio, WL01372).

### 4.25 Cell Culture and Phenotype Analysis

Rat bone marrow‐derived mesenchymal stem cells (BMSCs) were isolated and cultured in Dulbecco's Modified Eagle's Medium (DMEM, Servicebio, G4510) supplemented with 1% fetal bovine serum (FBS, TIANHANG, 11011‐8611) at 37°C in a 5% CO_2_ atmosphere. The cellular morphology was examined using an inverted microscope. BMSCs at the third passage were utilized for further analysis. Phenotypic characterization was conducted via flow cytometry, assessing the expression of CD90, CD105, CD73, CD45, and CD11b. After an incubation period of 8 days, the BMSCs were collected for subsequent MTT and cell cycle assays.

### 4.26 Cell Proliferation Assays

Cell proliferation assays were conducted utilizing the MTT assay. A range of 2000 to 8000 cells was seeded into 96‐well plates and incubated at 37°C. The relative cell numbers were assessed by staining the cells with MTT dye at specified time intervals. Subsequently, the stained cells were solubilized using an SDS‐dimethylformamide solution, and the absorbance was measured at 490 nm using a spectrophotometer.

### 4.27 Cell Cycle Assays

A cell cycle detection kit (Wanleibio, WLA010a) was utilized to assess the cell cycle dynamics. Cells were harvested and fixed in 70% ethanol for a duration of 24 h. The fixative was subsequently removed by washing with phosphate‐buffered saline (PBS). Thereafter, 100 μL of RNaseA was added to the cell pellet and incubated in a water bath at 37°C for 30 min. Following this incubation, 400 μL of propidium iodide (PI) was introduced, and the mixture was allowed to incubate for an additional 30 min. The cell cycle distribution was then analyzed using flow cytometry.

### 4.28 Antiapoptosis Assays

Cell apoptosis assays were performed using the Annexin V‐PI Apoptosis Detection Kit I (Wanleibio, WLA001a) following the manufacturer's protocol. Flow cytometry analysis was subsequently carried out using a Beckman instrument (USA).

### 4.29 Real Time‐PCR

RNA was extracted from tissue samples using TRIzol (BioTeke, RP1001) and converted to cDNA with the BeyoRT II M‐MLV kit (Beyotime, D7160L) after purification. For PCR, a mixture of 1 μL cDNA, 0.5 μL each of upstream and downstream primers, and 10 μL SYBR GREEN master mix was prepared, adjusted to 20 μL with water. The PCR conditions were: 95°C for 3 min, followed by 35 cycles of 94°C for 15 s, 56°C for 30 s, and 70°C for 60 s, ending with a 4°C hold for 5 min. Data were analyzed using a fluorescence quantitative analyzer, and gene expression levels were calculated with the 2^−△△*Ct*^ method. Primer/probe sets for rat genes are listed in Table S1.

### 4.30 Microcomputed Tomography (μCT)

The specimens were scanned with the Bruker Micro‐CT Skyscan 1276 system using a 6.534165 μm voxel size, medium resolution, 70 kV, 200 μA, a 0.25 mm aluminum filter, and a 350 ms integration time. Density was calibrated using the manufacturer's CaHA phantom. Analyses were performed with the manufacturer's software, and images were reconstructed with NRecon (v1.7.4.2). Three‐dimensional images were created from two‐dimensional ones using CTvox (v3.3.0), and both 3D and 2D analyses were done with CT Analyzer (v1.20.3.0). Bone microstructure indices, including bone volume fraction (BV/TV), trabecular number (Tb.N), and trabecular separation (Tb.Sp), were quantified using the Skyscan system.

### 4.31 Statistical Analysis

All analyses were performed using GraphPad Prism version 10.0 (GraphPad Software Inc., San Diego, CA, USA). Multiple comparisons were performed using one‐way analysis of variance (ANOVA) followed by Tukey's post hoc test or Bonferroni's post hoc test. Data depicted in bar charts are presented as the mean ± standard deviation. Box‐and‐whisker plots illustrate the range from the first quartile (the lower whisker) to the third quartile (the upper whisker), with a line within the box representing the median. Animals were randomly assigned to different experimental groups, and no animals or data points were excluded from the analyses for any reason. Results were considered statistically significant when *p*‐values were less than 0.05. All statistical tests, sample sizes and their *p* values are now provided in the figures and corresponding legends.

## Author Contributions

Conceptualization was developed by H.‐S.C. J.‐R.C. and Y.‐X.N. conducted the D‐gal‐induced aging model. J.‐R.C. and J.Z. performed periodic therapeutic phlebotomy. All behavior tests were accomplished by K.‐X.W. and T.‐C.X. J.‐R.C. and J.Z. did the immunofluorescence imaging and immunohistochemistry imaging and analysis. Flow cytometry analysis was accomplished by J.‐R.C. and Y.‐X.N. Electrophysiology‐related experiments were accomplished by J.‐R.C. and J.Z. The original draft was written by J.‐R.C., J.Z., M.‐C.L., and T.‐C.X. Review and editing were carried out by all authors. H.‐S.C. critically revised the manuscript. The project was supervised by H.‐S.C.

## Funding

The work was supported by grants from the National Natural Science Foundation of China (No. 82071477), the Science and Technology Project Plan of Liao Ning Province (2024JH6/100700015), and the Science and Technology Project Plan of Shenyang (20‐205‐4‐007).

## Conflicts of Interest

The authors declare no conflicts of interest.

## Supporting information


**Appendix S1:** Supporting Information.

## Data Availability

The data that support the findings of this study are available on request from the corresponding author. The data are not publicly available due to privacy or ethical restrictions.
