# Supplementary material for: Periodic Therapeutic Phlebotomy Mitigates Systemic Aging Phenotypes by Promoting Bone Marrow Function
Source: Aging Cell. 2026 Feb 2;25(2):e70400. doi: 10.1111/acel.70400 (PMC12862434; doi:10.1111/acel.70400)
Supplement: Supplementary file 1 — Appendix S1: Supporting Information. [file ACEL-25-e70400-s001.docx]

**Supplemental Table 1. Primers used in quantitative real-time PCR assay**

| **Gene** | **Forward primer (5’-3’)** | **Reverse primer (5’-3’)** |
| --- | --- | --- |
| Casp8 | GAAGGAAGCCTCTATCTATG | CCTGTTCTAAGCCTGTCTC |
| Cdkn1a | CAAAGTATGCCGTCGTCTGT | CAAAGTTCCACCGTTCTCG |
| Cdkn2a | TGATGGGCAACGTCAAAGT | TCGTGATGTCCCCGCTCT |
| IL-1b | TGTGATGTTCCCATTAGAC | AATACCACTTGTTGGCTTA |
| PAI1 | CCGCCTCCTCATCCTGCCTAA | AGACGCCACTGTGCCGCTCT |
| Tnfa | GTAGCAAACCACCAAGCG | GGTATGAAATGGCAAATCG |
| p21 | GAGAACTGGGGAGGGCTTTC | TCCTGAGCCTGTTTCGTGTC |
| IL-6 | AGTTCCGTTTCTACCTG | TCCTTAGCCACTCCTT |
| β-actin | GGAGATTACTGCCCTGGCTCCTAGC | GGCCGGACTCATCGTACTCCTGCTT |

**Supplemental figures 1-6:**

**
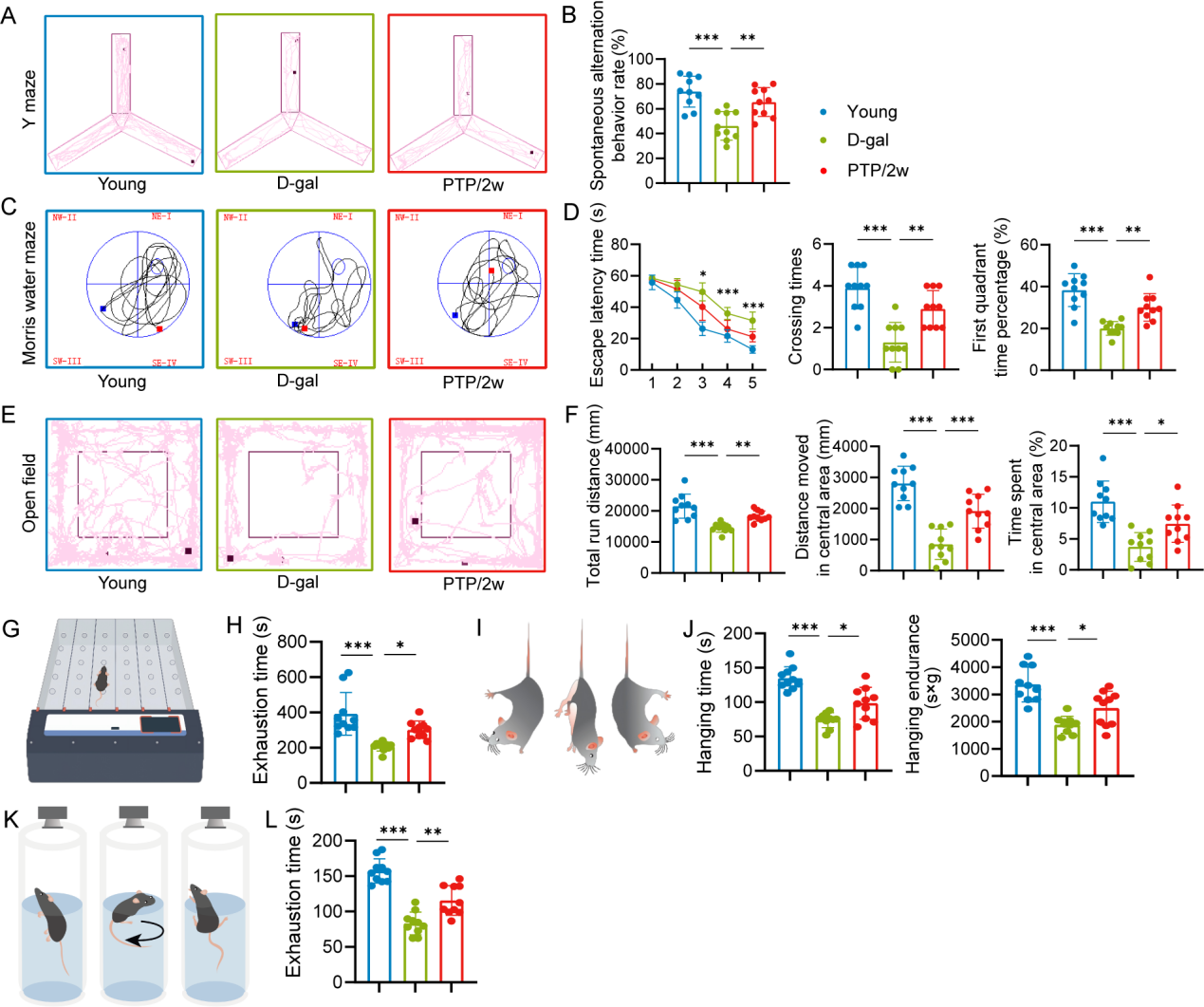
**

**Figure S1. PTP improves cognitive and motor performance.**

(A) Representative moving trajectory in Y maze of mice. (B) Spontaneous alternation behavior rate of mice in Y maze. (C) Representative moving trajectory in Morris water maze of mice. (D) Escape latency time, crossing times and first quadrant time percentage in Morris water maze. (E) Representative moving trajectory in open field. (F) Mice total run distance, distance moved, time spent in central area of open field. (G) Diagram of treadmill. (H) Exhaustion time of mice treadmill. (I) Diagram of tail suspension test. (J) Hanging time and hanging endurance of mice tail suspension test. (K) Diagram of forced swim test. (L) Exhaustion time of mice forced swim test. n=10/group. Statistical analysis: two way ANOVA and Tukey post-test for (D) escape latency time and one way ANOVA and Tukey post-test for others. Data are mean ± SD; error bars denote 95% C.I.; *p < 0.05, **p < 0.01, ***p < 0.001.

**
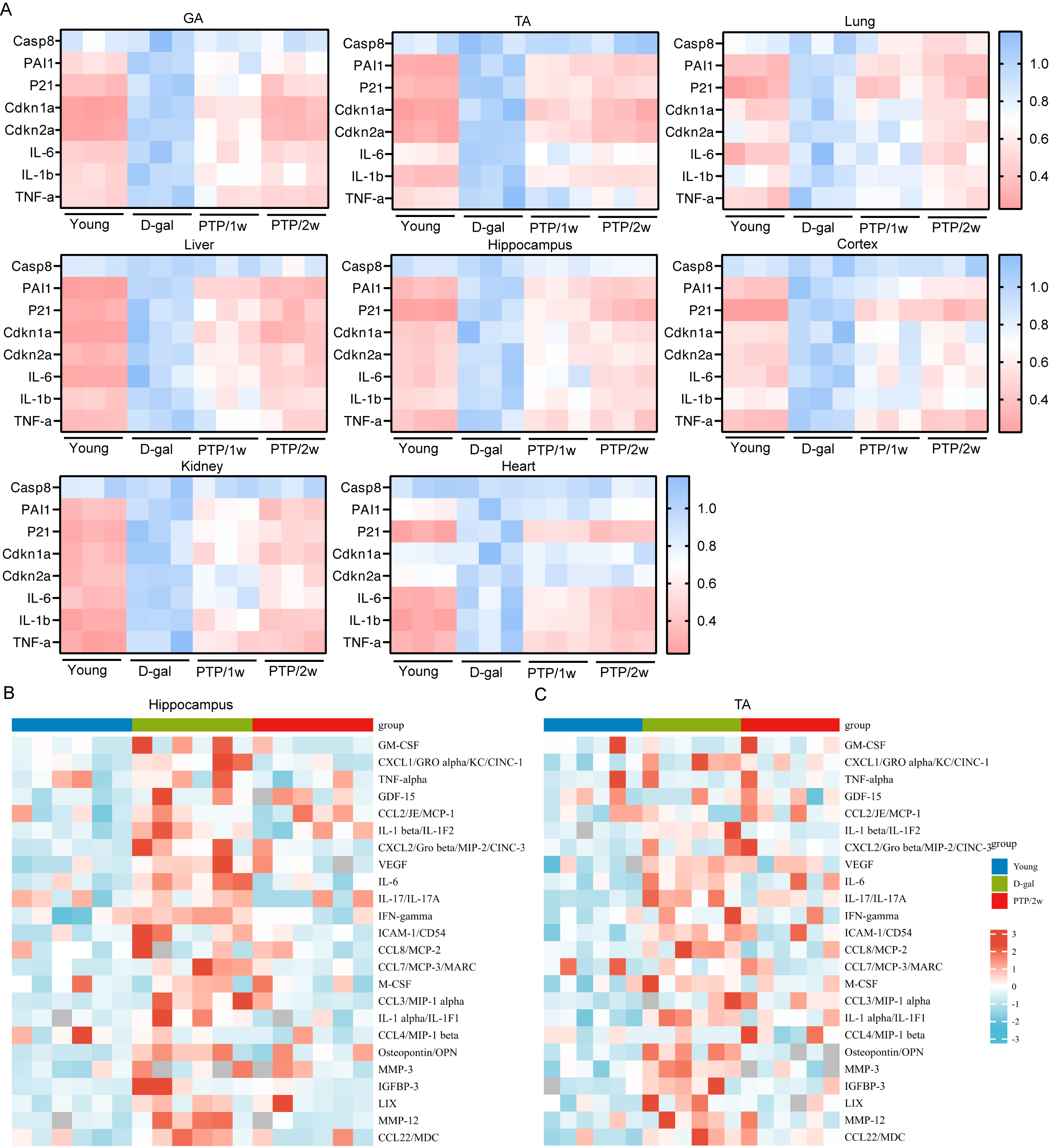
**

**Figure S2a. PTP reverses the senescence of tissues and organs.**

1. Gene expression of SASP markers in rat skeletal muscle (gastrocnemius (GA) and tibialis anterior (TA)), lung, liver, hippocampus, cortex, kidney, and heart, n =3/group. (B-C) Multiple cytokine profiling quantification of SASP proteins, normalized to the D-gal group, n=5-6/group, using the Luminex™ multiplex assays,in (B) murine hippocampus and (C) tibialis anterior. SASP senescence-associated secretory phenotype. The analysis was conducted using R software version 4.2.1 and the ComplexHeatmap package version 2.13.1, employing a Z-score transformed data matrix with row normalization.

**
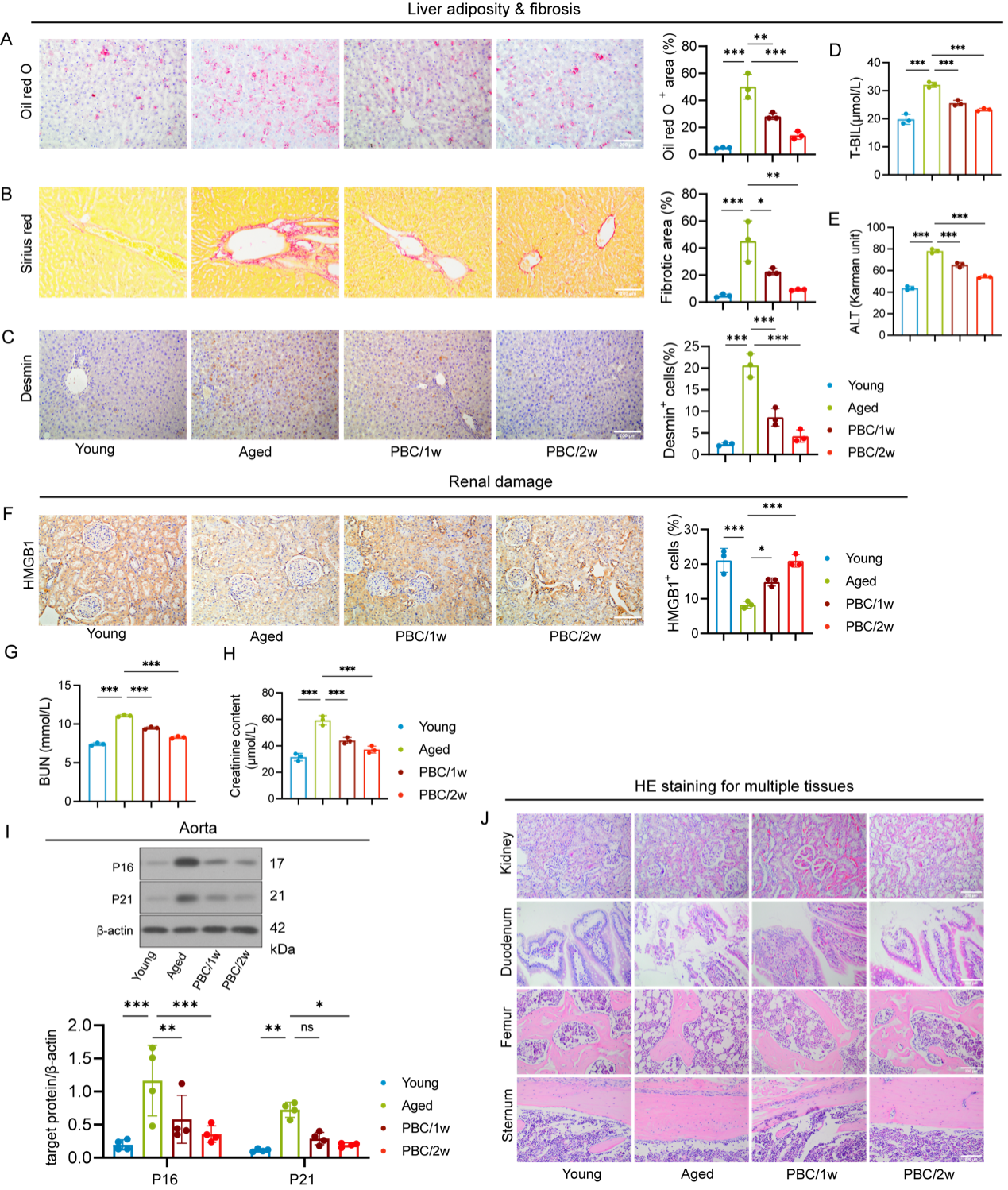

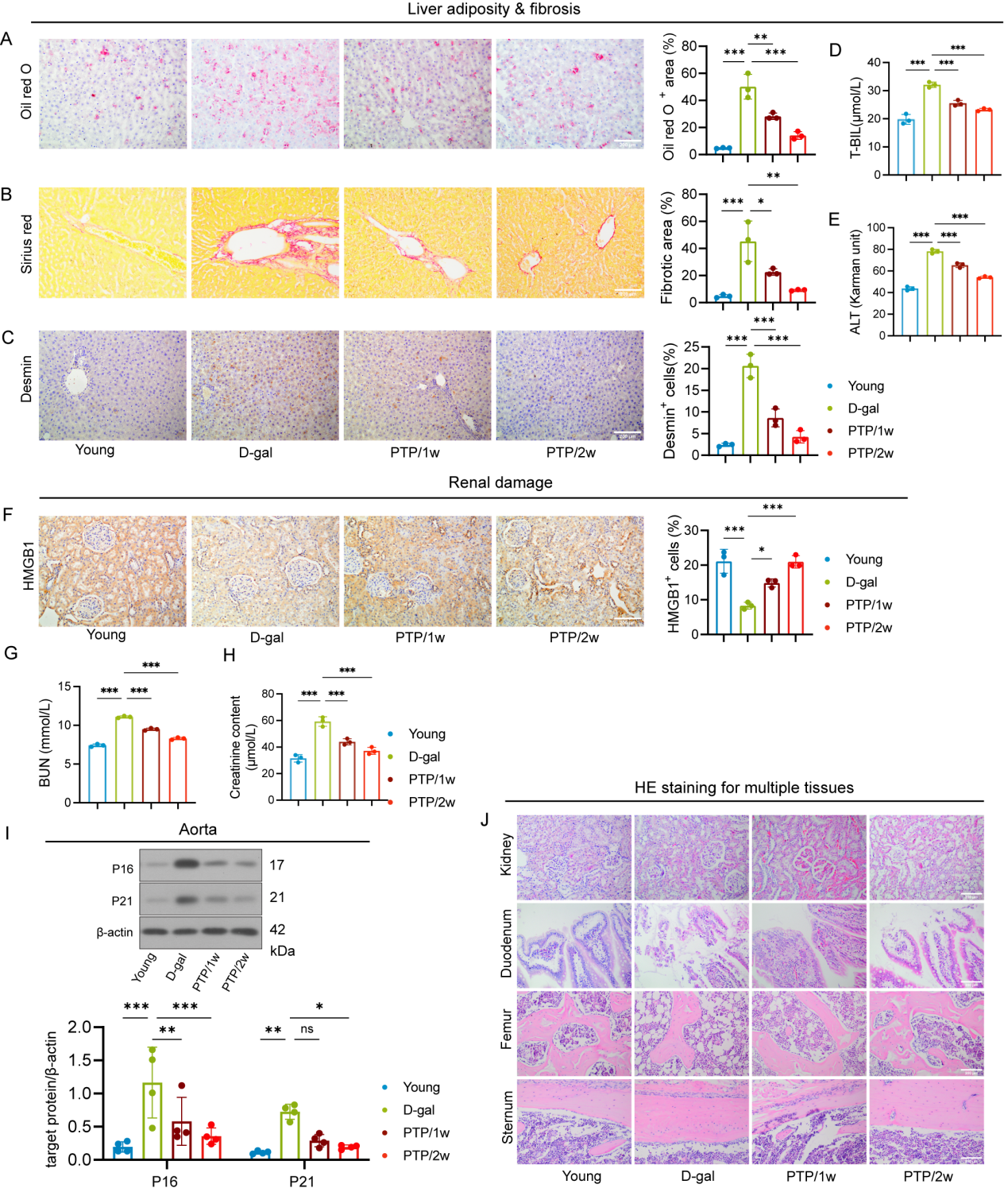
**

**Figure S2b. PTP reverses the senescence of tissues and organs.**

1. C) Representative Oil red O, Sirius red staining and Desmin immunohistochemistry of rat liver (scale bars, 200 μm), and quantification of Oil red O^+^ area, fibrotic area, and Desmin^+^ cells, n=3/group. (D, E) T-BIL (μmol/mL) and ALT (Karman unit) in rat peripheral blood serum, n=3/group. (F) Representative staining of HMGB1 immunohistochemistry of rat kidney (scale bars, 200 μm), and quantification of HMGB1^+^ cells, n=3/group. (G, H) Quantification of BUN (μmol/mL) and creatinine content (μmol/mL) in rat peripheral blood serum, n=3/group. (I) Western blot and quantification of P16, P21 expression in rat aorta, n=4/group. (J) Representative hematoxylin and eosin (HE) staining of rat kidney, duodenum, femur, and sternum (scale bars, 400 μm). T-BIL total bilirubin, ALT alanine aminotransferase, BUN blood urea nitrogen. Statistical analysis: one way ANOVA and Tukey post-test. Data are mean ± SD; error bars denote 95% C.I.; *p < 0.05, **p < 0.01, ***p < 0.001.

**
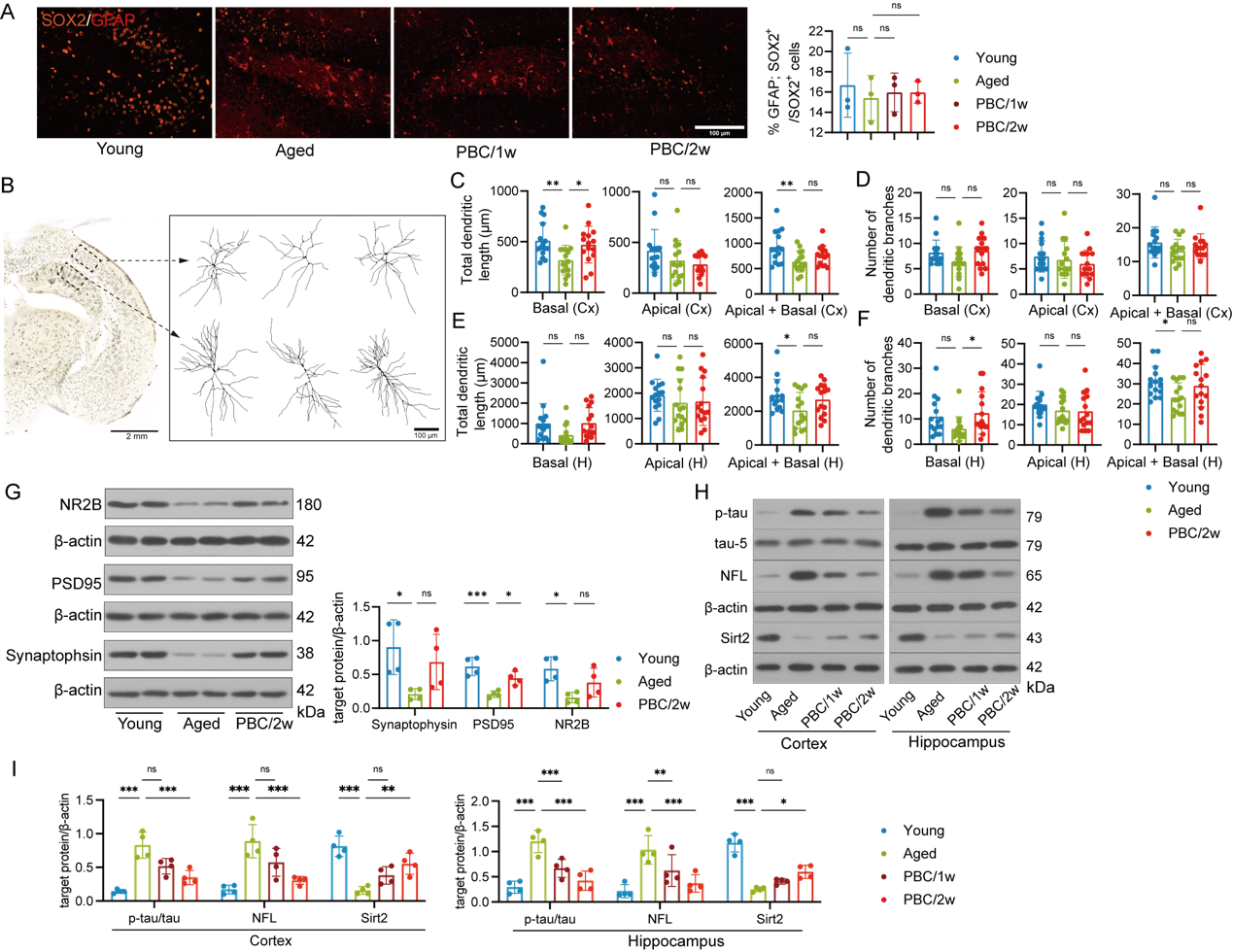

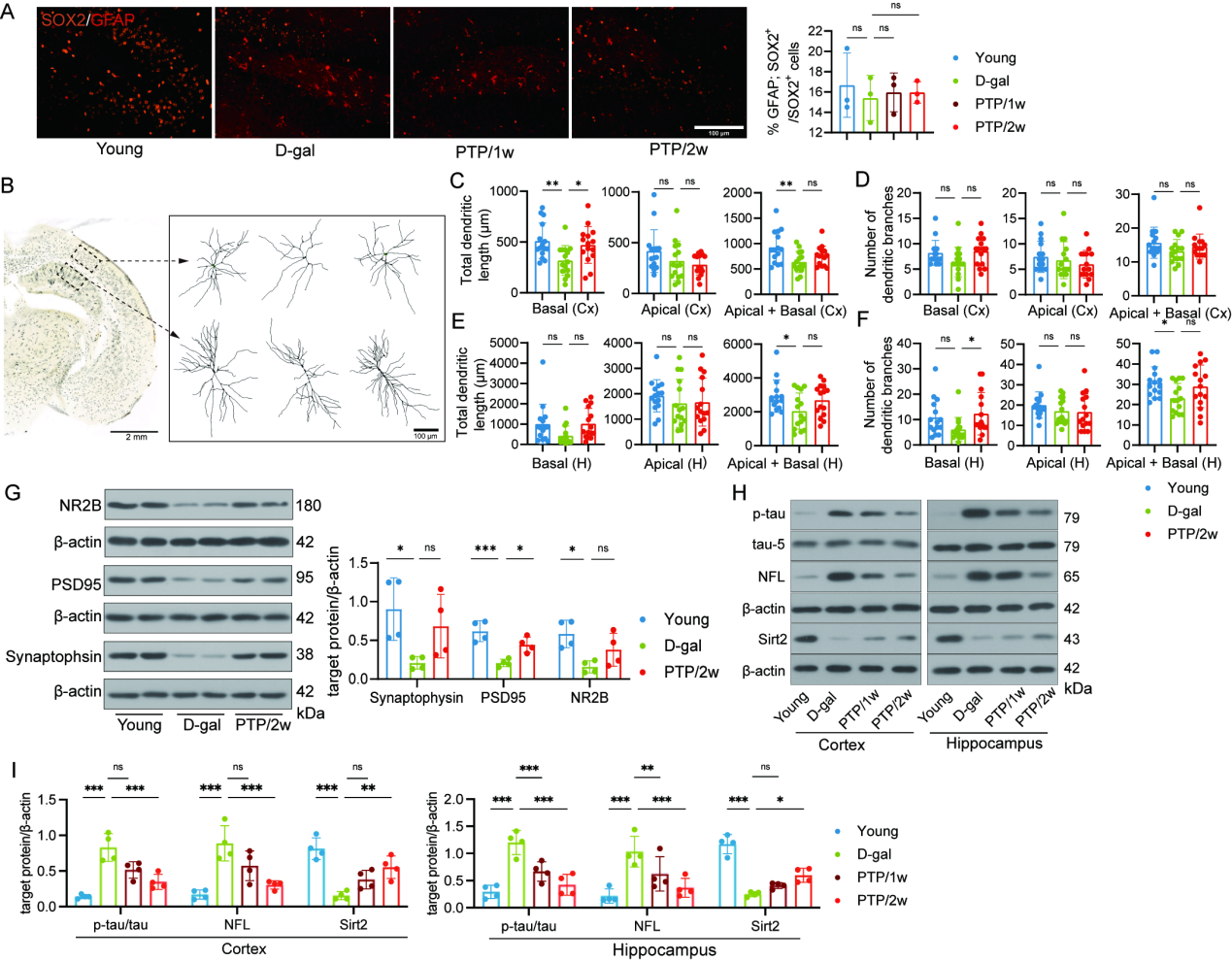
**

**Figure S3. PTP intervention mitigates the decline in neurogenesis and excitability of neurons.**

(A) Representative immunofluorescence staining of SOX2 and GFAP in hippocampus coronal subgranular zone (SGZ) of rats (scale bars, 100 μm), and quantification of SOX2^+^GFAP^+^ cells, n=3/group. (B) Representative Golgi staining of cortical and hippocampal neurons from mice. Scale bars, 2 mm for low magnified views and 100 μm for high magnified views. (C) Total dendritic length in basal and apical part of mice cortex, 15 neurons from 3 samples. (D) Number of dendritic branches in basal and apical part of mice cortex, 15 neurons from 3 samples. (E) Total dendritic length in basal and apical part of mice hippocampus, 15 neurons from 3 samples. (F) Number of dendritic branches in basal and apical part of mice hippocampus, 15 neurons from 3 samples. (G) Western blot and quantification of NR2B, PSD95 and synaptophysin in cortex and hippocampus of mice, n=4/group. (H) Western blot of p-tau, tau-5, NFL, and Sirt2 in cortex and hippocampus of rats. (I) Quantification of p-tau/tau, NFL, and Sirt2 expression, n=4/group. Statistical analysis: one way ANOVA and Tukey post-test. Data are mean ± SD; error bars denote 95% C.I.; *p < 0.05, **p < 0.01, ***p < 0.001.

**
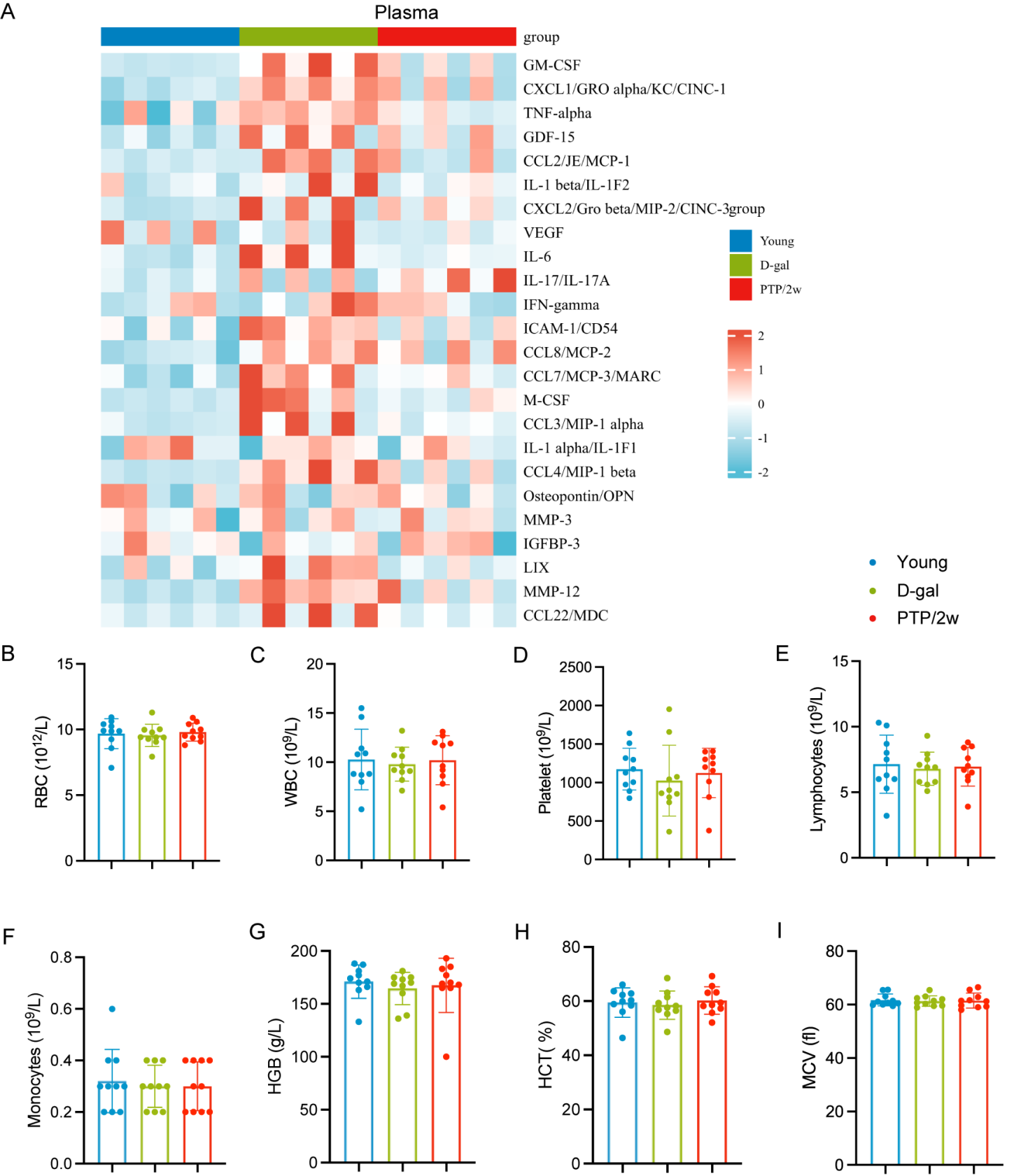
Figure S4. PTP mediates the reversal of overall aging through the rejuvenation of peripheral blood.**

(A) Multiple cytokine profiling quantification of SASP proteins in mice peripheral blood plasma normalized to the D-gal group, n=6/group, using the Luminex™ multiplex assays. (B) RBC (10^9^/L), (C) WBC (10^9^/L), (D) Platelet (10^9^/L), (E) Lymphocytes (10^9^/L), (F) Monocytes (10^9^/L), (G) HGB (g/L), (H) HCT (%), (I) MCV (fl) were detected through the blood routine test in peripheral blood of rats, n=10/group. WBC white blood cell, RBC red blood cell, HGB hemoglobin, HCT hematocrit, MCV mean corpuscular volume. Statistical analysis: one way ANOVA and Tukey post-test. Data are mean ± SD; error bars denote 95% C.I.; *p < 0.05, **p < 0.01, ***p < 0.001.

**
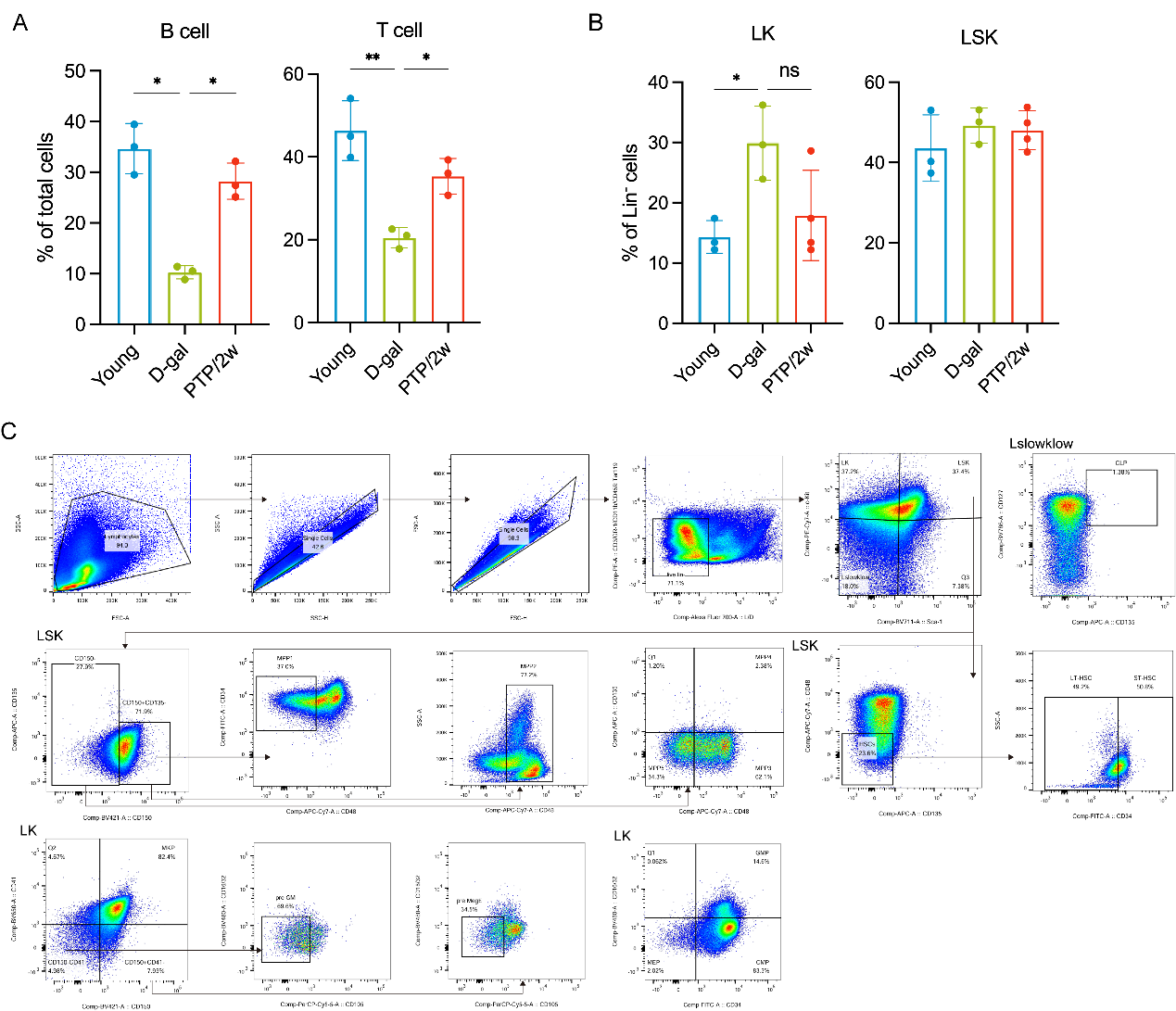
**

**Figure S5. PTP promotes the transformation of hematopoiesis to a youthful state by improving the hematopoietic microenvironment in the bone marrow.**

(A) B cell and T cell percentage of total cells from rat bone marrow were detected by flow cytometry, n=3/group. (B) LK and LSK cell percentage of lin^-^ cells from mice bone marrow were detected by flow cytometry, n=3-4/group. (C) Gating strategy and representative examples. LK lin^-^c-Kit^+^, LSK lin^-^sca1^+^c-Kit^+^. Statistical analysis: one way ANOVA, Bonferroni post-test for(B) and Tukey post-test for the others. Data are mean ± SD; error bars denote 95% C.I.; *p < 0.05, **p < 0.01, ***p < 0.001.
